# Sequencing Ultraconserved Elements (UCEs) for Marine Population Genomics: A Proof‐of‐Concept Using a Deep‐Sea Mussel Species

**DOI:** 10.1111/eva.70195

**Published:** 2026-01-13

**Authors:** Yi‐Xuan Li, Ting Xu, Maeva Perez, Chong Chen, Hiromi Kayama Watanabe, Jack Chi‐Ho Ip, Jian‐Wen Qiu

**Affiliations:** ^1^ Department of Biology Hong Kong Baptist University Hong Kong SAR China; ^2^ Department of Ocean Science The Hong Kong University of Science and Technology Hong Kong SAR China; ^3^ X‐STAR, Japan Agency for Marine‐Earth Science and Technology (JAMSTEC) Yokosuka Japan; ^4^ Science Unit, Lingnan University Hong Kong SAR China

**Keywords:** bivalves, deep sea, population genetics, pseudoreference, target capture

## Abstract

Ultraconserved elements (UCEs) have emerged as a powerful tool for resolving deep evolutionary relationships due to their low DNA quality requirements and broad taxonomic applicability. While their utility for intraspecific and shallow‐divergence studies is growing, only a few studies have explored their performance in marine taxa, some of them with metapopulations spanning thousands of kilometers. Here, we employed the UCE approach to investigate the population genomics of *Gigantidas platifrons*—a deep‐sea mussel with a long larval dispersal period that exhibits a panmictic genetic structure across its extensive distribution range in the chemosynthetic ecosystems of the Western Pacific. With its published whole genome and prior restriction site‐associated DNA sequencing using IIB restriction enzymes (2b‐RAD seq) study, this species is an excellent candidate for evaluating the effectiveness of UCEs. We conducted UCE target capture sequencing on 123 individuals collected from two hydrocarbon seeps and four hydrothermal vents, yielding 1960 UCEs. To assess the impact of different reference choices, we identified 11,870 single‐nucleotide polymorphisms (SNPs) by mapping against the published genome and 8936 SNPs by mapping to the representative 1960 UCEs. Both datasets were similar, with over 80% of the SNPs located in intronic and intergenic regions. Analyses based on both datasets consistently implied a clear genetic divergence between the South China Sea (SCS) and Okinawa Trough‐Sagami Bay (OT‐SB) populations, with predominant gene flow from OT to SB, consistent with previously published 2b‐RAD seq findings. Additionally, UCE‐based SNPs identified a dynamic decline in population size for individuals in the three regions and revealed selective adaptation signals to their environments. Overall, our study serves as a proof‐of‐concept demonstrating that UCEs provide a comparable resolution to RAD‐Seq in detecting shallow‐level genetic divergence and delineating conservation units in a high‐dispersal marine species, even when lacking a sequenced genome.

## 1 Introduction

Population genetics provides insights into changes in the allele frequencies within and between populations across space and time. It is urgent to understand the population connectivity of organisms as global climate change and human activities reduce the genetic diversity of many natural populations (Stange et al. 2020; Hohenlohe et al. 2021). Allelic changes typically include single‐nucleotide polymorphisms (SNPs), short insertions/deletions (indels), and structural variants; among them, SNPs have been most frequently used to study the population‐level genetic divergences among natural populations (Dokan et al. 2021). Advances in sequencing technologies have enabled the generation of SNP datasets for population genetic studies through whole genome resequencing, restriction‐site‐associated DNA (RAD‐Seq), target enrichment methods such as Anchored Hybrid Enrichment (AHE), or transcriptome sequencing (Lemmon et al. 2012; Crawford and Oleksiak 2016). Another target capture approach, ultraconserved elements (UCEs) enrichment sequencing, is a promising tool. UCEs—highly conserved genomic regions shared across distant evolutionary taxa—have been mainly used to resolve challenging deep phylogenetic relationships (e.g., McCormack et al. 2011; Faircloth et al. 2012; Harvey et al. 2016; Chen et al. 2018; Derkarabetian et al. 2019). Since the flanking regions of UCEs contain abundant variations, Faircloth et al. (2012) proposed that they could be useful for studies even at shallow evolutionary timescales.

Empirical analyses have demonstrated that UCEs provide comparable to (Harvey et al. 2016) or even superior resolution to RAD‐Seq (Glon et al. 2021) for delimiting cryptic species and detecting population genetic structure. RAD‐Seq utilizes restriction enzyme digestion to obtain SNP markers, advanced in low cost and high throughput, but were limited by low coverage, paralog confusion, and bias from uneven restriction site distribution (Vendrami et al. 2017). One of the key advantages of UCEs is that they can be used across broad taxonomic scales due to their ultra‐high conservation, facilitating comparisons among taxonomic groups and studies, whereas RAD‐Seq markers are species‐specific. UCEs' compatibility with low‐quality or historic samples (Byerly et al. 2024), combined with their broad taxonomic applicability, makes them a versatile tool for studying population genetics across diverse marine taxa. Several studies have highlighted the effectiveness of UCEs in discriminating cryptic species and assessing population connectivity in vertebrates, including sea dragons (Stiller et al. 2021), amphibians (Chan et al. 2020), snakes (Myers et al. 2019), and birds (Harvey et al. 2016; Zarza et al. 2016; Winker et al. 2018; Mapel et al. 2021). However, applications of UCEs in marine invertebrates are limited and have only been carried out for a few anthozoans (Erickson et al. 2021; Glon et al. 2021; Gordon 2023), two polychaetes, an amphipod, and two bivalve species (Petersen, Hansen, et al. 2022; Petersen, Knott, et al. 2022). Two studies on cnidarians reported that 152–2742 UCE‐captured SNPs can successfully delimitate genetic structure (Erickson et al. 2021; Glon et al. 2021). While Petersen, Hansen, et al. (2022) and Petersen, Knott, et al. (2022) applied 1–29 UCEs shared among samples for six taxa, they failed to detect clear population structure due to limited data. Although over 1000 SNPs may be sufficient for identifying genetic structure without false positives (Aguirre‐Liguori et al. 2020), the optimal SNP number for marine taxa remains untested (Petersen, Knott, et al. 2022).

Although a high‐quality genome is ideal for SNP calling (Theissinger et al. 2023; Thorburn et al. 2023), a large number of invertebrate species lack a sequenced genome. For instance, in the phylum Mollusca, only 283 out of over 60,000 extant species have a sequenced genome (Sigwart et al. 2021; Aristide and Fernández 2023; Challis et al. 2023). The scarcity of reference genomes is typically overcome by using consensus sequences generated through user‐friendly pipelines, such as SqCL (Harvey et al. 2016; Singhal et al. 2017). However, there is a lack of comprehensive comparisons regarding the consequences of reference choice, such as the effectiveness of complete genomes versus UCE‐based pseudoreferences. Recent UCE‐focused studies have employed various reference sources for SNP calling, extending beyond captured UCEs (Singhal et al. 2017; Stiller et al. 2021; Zhang, Zeng, et al. 2021; Petersen, Knott, et al. 2022; Ortiz‐Sepulveda et al. 2023), including the complete genome (Thacker et al. 2022) and mega‐assemblies from individuals (Lim and Braun 2016; Winker et al. 2018; Lim et al. 2020). Notably, the reference quality may affect the variant detection outcomes, which remain an unresolved issue in practice and merit further investigation to enhance the reliability and accuracy of SNP calling.

Here, we aim to compare the efficacy of UCEs and RAD‐Seq in population genetic studies of bivalves. Specifically, we examined whether a set of UCEs developed for Bivalvia (Li et al. 2025)—a major class of Mollusca with fossil records extending to the Early Cambrian—can be applied to population genetics. We selected *Gigantidas platifrons* (previously *Bathymodiolus platifrons*) (Mytilidae, Bivalvia) as the test organism due to its wide horizontal and vertical distribution in deep‐sea hydrothermal vents and methane seeps across the Northwest Pacific (642–1684 m) and previous studies showing genetic admixture regarding this species' population genetic structure and gene flow (Watanabe et al. 2010; Zhao et al. 2020). Based on allelic variation in one to several mitochondrial genes, three studies revealed no genetic differentiation among the populations spanning from the South China Sea (SCS) to Sagami Bay, suggesting panmixia across this species' entire distribution range (Kyuno et al. 2009; Miyazaki et al. 2013; Shen et al. 2016). However, using genome‐wide SNPs generated by the 2b‐RAD approach (a form of RAD‐Seq), Xu et al. (2017, 2018) detected significant genetic divergence and gene flow among the populations, especially between the Okinawa Trough (OT) and a methane seep in the SCS populations, highlighting the importance of choosing markers with sufficient resolution for population structure and gene flow analyses. Therefore, *G. platifrons* is a good candidate to test whether the UCEs developed for Bivalvia can be successfully applied to answer evolutionary questions at shallow timescales and present a resolution comparable to RAD‐Seq. Given that *G. platifrons* has a sequenced genome (Sun et al. 2017), we also aimed to compare the efficacy of SNPs obtained by mapping to the genome and the pseudo‐reference generated by pooling UCEs.

## 2 Materials and Methods

### 2.1 Sample Collection and DNA Extraction

**FIGURE 1:**
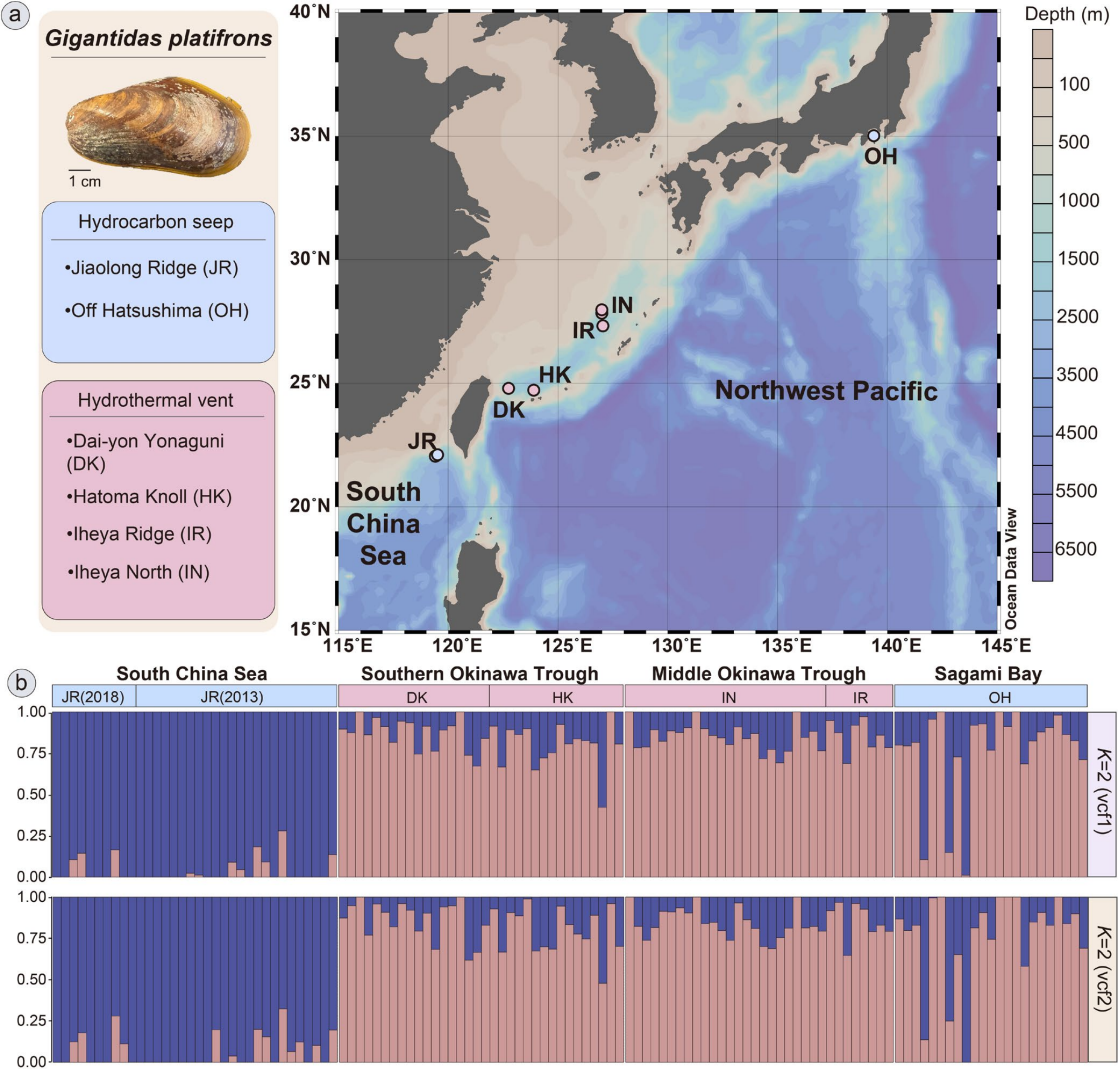
Geographic and genetic populations of *Gigantidas platifrons* in the Northwest Pacific. (a) Sampling map; (b) admixture structure analysis result. M‐OT, Middle Okinawa Trough; Ref1, *G. platifrons* genome; Ref2, Pseudoreference; SB, Sagami Bay; SCS, South China Sea; S‐OT, Southern Okinawa Trough. Blue color, hydrocarbon seep region; pink color, hydrothermal vent region.

To compare with a previous population genomic study using the same species (Xu et al. 2018), we adopted almost the same specimens and their extracted DNA samples (Table S1). If the leftover DNA samples passed quality control, they were used for UCE library preparations, as detailed in the following section. Otherwise, the original tissue was used to re‐extract DNA. In addition to the 101 specimens used by Xu et al. (2018), we included 22 specimens to increase the sample size (Table 1). In brief, a total of 123 *Gigantidas platifrons* samples were collected from two hydrocarbon seeps (57 individuals) and four hydrothermal vents (66 individuals) in the Western Pacific during nine dives between 2011 and 2018 (Figure 1; Tables 1 and S1; Xu et al. 2018). These sites are located across four geographic regions: the South China Sea (SCS, seep), Southern Okinawa Trough (S‐OT, vent), Middle Okinawa Trough (M‐OT, vent), and Sagami Bay (SB, seep). Samples were preserved in absolute ethanol at −20°C or frozen immediately at −80°C upon arrival at the main deck of the respective research vessel. Genomic DNA was extracted from the adductor muscle of each individual using the phenol/chloroform extraction method, qualified and quantified by 1.5% agarose gel electrophoresis, NanoDrop^@^ 2000 Spectrophotometers (Thermo Fisher Scientific, MA, USA), and by Qubit 4 fluorometer using Qubit^@^ dsDNA HS Assay Kits (Thermo Fisher Scientific, MA, USA).

**TABLE 1:** Basic sample information of *Gigantidas platifrons* with more details included in Table S1.

Region	Location	Code	Habitat	Depth (m)	Collection year	Number	Xu et al. (2018)
South China Sea	Jiaolong Ridge	JR	Seep	1122	2018	10^a^	0
South China Sea	Jiaolong Ridge	JR	Seep	1122	2013	24	30
Sagami Bay	Off Hatsushima	OH	Seep	1172	2011	12	12
Sagami Bay	Off Hatsushima	OH	Seep	858	2009	11	12
Southern Okinawa Trough	Hatoma Knoll	HK	Vent	1482	2014	16 (1^a^)	15
Southern Okinawa Trough	Dai‐yon Yonaguni Knoll	DK	Vent	1344	2014	18	20
Middle Okinawa Trough	Iheya North	IN	Vent	993	2013	19 (11^a^)	8
Middle Okinawa Trough	Iheya North	IN	Vent	1002	2014	3	3
Middle Okinawa Trough	Iheya Ridge	IR	Vent	1402	2011	10	10

^a^ Specimens used only in this study.

### 2.2 Library Preparation, Target Capture Enrichment, and High‐Throughput Sequencing

The genomic DNA was physically sheared using a Diagenode Bioruptor Pico sonication device (Diagenode, CA, USA), and electrophoresis was run on 1.5% agarose gel to determine the fragment size distribution. The sheared DNA was then used for library preparation, including end‐repair, A‐tailing, adapter ligation, and amplification using the Invitrogen Collibri PS DNA Library Prep Kit for Illumina Systems, with UD indexes (Thermo Fisher Scientific, MA, USA) applied to provide a unique dual index for each library. Each purified adaptor‐ligated DNA library was amplified through polymerase chain reactions (PCRs) with 2–16 cycles according to its quantity. Each library was quantified by a Qubit 4 fluorometer using Qubit^@^ dsDNA HS Assay Kits, with size distribution determined by 1.5% agarose gel electrophoresis.

Due to variations in DNA quality among samples, we pooled samples with similar DNA quantities and quality, targeting 250 ng per sample as the starting material. Ten or 12 equimolar samples were pooled and then speed‐vacuumed to a final volume of 7 μL, following the method described by Li et al. (2025). The target enrichment capture was conducted using a MYbaits Kit, aiming to capture ~2000 UCEs from the coding and non‐coding regions. The probe set Bivalve UCE 2k v.1 (20,000 probes) was designed by Li et al. (2025) with available molluscan genomes and transcripts (including *G. platifrons*). The *in silico* and in vitro recovery results of *G. platifrons* UCEs were detailed in Table S2. The target capture was conducted according to the MYbaits Kit User Manual v.5.01, except for the hybridization, which was conducted at 60°C for 21–24 h. The final clean PCR‐amplified hybrid libraries were quantified using a Qubit 4 fluorometer and qPCR, and the peak size was quantified using a 2100 Bioanalyzer (Agilent, CA, USA). The qualified pools, with 0.45–2.08 nmol/L qPCR concentration and fragment peak size at around 544–580 bp, were sequenced using an Illumina Novaseq 6000 platform to generate 150‐bp paired‐end reads at Novogene (Tianjin, China).

### 2.3 Data Processing and Construction of Reference Databases

The raw reads of each sample were demultiplexed based on their unique dual index and further processed using PHYLUCE v.1.7.2 (Faircloth 2016). The raw reads of each individual were trimmed to remove the adaptors and low‐quality bases using Illumiprocessor v.2.0.9 (Faircloth 2013; Bolger et al. 2014) under default settings.

To detect variants, we first constructed reference databases using the genome (Ref1) of *G. platifrons* (Bioproject accession number: PRJNA328542; Sun et al. 2017) and pseudoreference (Ref2), representative UCEs generated from all individuals. The three essential Ref1 index databases were created using BWA‐MEM2 v.2.2.1 (Li 2013) with the function “index,” Picard v.2.27.5 (BroadInstitue 2019) with the function “CreateSequenceDictionary,” and samtools v.1.19 (Danecek et al. 2021) with the function “faidx,” respectively. To create our new “pseudoreference” (Ref2), we assembled clean data of each sample using SPAdes v.3.15.5 (Nurk et al. 2013) and constructed a consensus reference from all assemblies using the SqCL script “make_PRG.py” (Singhal et al. 2017). The non‐redundant Ref2 contigs were filtered using cd‐hit‐est v.4.8.1 (Fu et al. 2012) with “‐c” set to 0.98, and then checked using QUAST v.5.2.0 (Mikheenko et al. 2018). These filtered contigs were subsequently applied to construct index databases, as for Ref1. Ref2 was annotated by BLASTn against the *G. platifrons* genome (Sun et al. 2017) and filtered by a cutoff of 80% identity. We also conducted functional annotation of Ref2 using eggNOG‐mapper v.2 under default settings (Cantalapiedra et al. 2021). The composition of Ref2 was visualized using Chiplot (https://www.chiplot.online/).

### 2.4 SNP Identification and Filtering

For each reference, we performed mapping using BWA‐MEM2 v.2.2.1 and samtools v.1.19, followed by removing duplicates using Picard v.2.27.5. The filtered mapping reads were summarized by applying samtools “flagstat” and “coverage.” We conducted variant calling analysis using the “HaplotypeCaller” in GATK v.4.1 (McKenna et al. 2010), combined all gvcf using “CombineGVCFs,” genotyped variants using “GenotypeGVCFs,” and selected SNPs using “SelectVariants.”

Further filtering of SNPs was carried out with the following criteria: (1) filtering low‐quality SNPs using GATK “VariantFiltration” with criteria of “QD < 2.0 || MQ < 40.0 || FS > 60.0 || SOR > 3.0 || MQRankSum < −12.5 || ReadPosRankSum < −8.0” and “SelectVariants—exclude‐filtered,” (2) only keeping biallelic SNPs using GATK “SelectVariants—restrict‐alleles‐to BIALLELIC,” (3) removing SNPs with a depth lower than 10× for each individual or mean depth higher than 20× over all included individuals (settings: –minDP 10 –max‐meanDP 200) using vcftools v.0.1.16 (Danecek et al. 2011), (4) removing SNPs with missing data using vcftools, (5) removing SNPs with minor allele frequency (MAF) < 0.05 using vcftools, and (6) removing SNPs for each population with *p* < 0.01 in the Hardy–Weinberg equilibrium test using the “filter_hwe_by_pop.pl” script (Puritz et al. 2014). The filtered SNP datasets from Ref1 and Ref2 named vcf1 and vcf2, respectively, were then used for downstream analysis. The depth at each base for each individual, as well as the mean sequencing depth per individual, was calculated using samtools. Two additional filtered datasets (vcf3 and vcf4) were generated from Ref1 and Ref2 to estimate genetic diversity statistics. SNPs in vcf3 and vcf4 were thinned to one SNP per 10,000 bp or per locus using vcftools, without applying the Hardy–Weinberg equilibrium filter. In addition, the invariant sites for vcf3 and vcf4 were retained using Pixy v.1 (Korunes and Samuk 2021) following the online tutorial (https://pixy.readthedocs.io/en/latest/generating_invar/generating_invar.html).

### 2.5 Genetic Statistics Estimation, Geographic Diversity, and Population Structure Analysis

Based on the filtered SNP datasets (vcf1 and vcf2), we calculated the genetic statistics of seven groups (Table 2), including observed heterozygosity (*H*_obs_) and expected heterozygosity (*H*_exp_) using vcftools, nucleotide diversity (*π*) using the script “popgenWindows.py” (https://github.com/simonhmartin/genomics_general), and *F*_ST_ using the R package hierfstat v. 0.5‐11 (Goudet 2005). Before *F*_ST_ calculation, the vcf files were imported to R using vcfR v.1.15.0 (Knaus and Grünwald 2017) and reformatted by adegenet v.2.1.9 (Jombart 2008). We explored the relationships between genetic divergence (*F*_ST_) and geographic distance, *F*_ST_ and vertical depth, and plotted the results using ggplot2 (Wilkinson 2011). To examine the correlation, Pearson correlation coefficients (r) and associated *p*‐value were calculated using SciPy v.1.0 with the “pearsonr” function (Virtanen et al. 2020).

**TABLE 2:** Information on the genome (Ref1, GCA_002080005.1) and representative UCEs from all individuals (pseudoreference Ref2).

	Ref1	Ref2
Number of scaffolds/contigs	65,664	1960
Total length of scaffolds/contigs (bp)	1,659,280,971	5,366,066
Longest scaffold/contig (bp)	2,790,175	9779
Average scaffold/contig (bp)	25,269	2737
GC (%)	34.17	33.5
N50 scaffold/contig length (bp)	345,903	2861
L50 scaffold/contig count (bp)	1256	719

To reveal the genetic structure among *G. platifrons* populations using vcf1 and vcf2, we applied PCA analysis using Plink v.1.9 (Purcell et al. 2007) and visualized results using ggplot2. Discriminant Analysis of Principal Components (DAPC) analysis was performed using adegenet v.2.1.9 with *K* = 4 (four sampling regions) and *K* = 7 (seven sampling sites). Admixture v.1.3.0 (Alexander and Lange 2011) was run to infer population genetic structure on the datasets with iterations over the cluster numbers of *K* = 2–4, and the results were plotted using pophelper v.2.3.1 (Francis 2017).

To facilitate comparison with the previous 2b‐RAD study (Xu et al. 2018), we estimated the genetic diversity statistics (*H*_obs_, *H*_exp_, and *π*) for seven groups (Table 2) using Pixy on the vcf3 dataset including invariant sites. In addition, we examined *F*_ST_ using Pixy and inferred population structure (PCA analysis) using Plink, based on the thinned datasets vcf3 and vcf4 for four regions (Table 2).

### 2.6 Demographic History Inferences

We used TreeMix v.1.13 (Pickrell and Pritchard 2012) to detect migrations between populations and regions based on the vcf1 and vcf2 datasets. The input for TreeMix was reformatted using Plink. The SCS group was set as the root of the other groups. The tree was constructed by testing one to five migration edges (i.e., “‐m,” m = 0–5) for four regions. To determine the optimal m, we performed TreeMix analysis with bootstrap *k* = 1000 and 12–15 iterations for each m, until no further iterations were required by OptM (Fitak 2021). The outputs were then evaluated using OptM. Besides the migration event detection, we estimated the demographic history of three groups (SCS, OT, and SB) using vcf1 and vcf2 by StairwayPlot2 (Liu and Fu 2020). The folded SNP frequency spectrum (SFS) for each group was generated using easySFS.py (Gutenkunst et al. 2009; https://github.com/isaacovercast/easySFS) with a projection size of 10 for each group. The stairway plot analysis was conducted under default settings with 1000 input file numbers, 1‐year generation time, and a mutation rate of 0.56 × 10^−8^ (deep‐sea species' mutation rate estimated between 0.09 × 10^−8^ and 0.56 × 10^−8^; Portanier et al. 2023) to estimate the recent effective population size changes (*N*e) for three groups, with results visualized using ggplot2.

### 2.7 Outlier SNP Identification and SNP Annotation

The candidate loci with outlier SNPs (sites with significantly different allele frequencies across populations) were identified between regions using three methods. First, we applied BayeScan v.2.1 (Foll and Gaggiotti 2008) for four regions (SCS, S‐OT, M‐OT, SB). The input files for BayeScan were reformatted using PGDspider v.2 (Lischer and Excoffier 2012). The analysis was conducted using two SNP datasets under default settings except for 10,000 burn‐in and a sample size of 1000, with a total of 20,000 iterations. The results of BayeScan analysis were plotted using R scripts provided by BayeScan with a false discovery rate (FDR) = 0.05. The outlier SNPs were selected using vcftools according to positions provided by BayeScan results. Furthermore, due to the geographic barrier between SCS and northern Pacific populations (Andres et al. 2015) and different environmental conditions between seeps and vents, we detected genomic regions with signatures of selection using *F*_ST_ and *π* ratio “Pi_seep/Pi_vent” between the seep group (SCS and SB) and vent group (OT) and “Pi_scs/Pi_pac” between the SCS and other Pacific groups (OT and SB). To prevent overly stringent or lenient selection under a uniform cutoff, we applied distinct quantile thresholds based on distribution characteristics. We selected genomic windows simultaneously with the high *π* ratio (top 10% for “Pi_seep/Pi_vent” and top 5% for “Pi_scs/Pi_pac”) and high *F*_ST_ value (above 0.05) of the empirical distribution as regions with strong selection signals along the reference genome. To check for symmetric changes, the relationship between log‐transformed “Pi_seep/Pi_vent” and “Pi_scs/Pi_pac” and *F*_ST_ was analyzed. The top low and high quantiles (5% for vcf1 and 15% for vcf2) were selected from the empirical distributions as regions with strong selection signals. These selected regions were merged and candidate loci under selection were extracted, referring to the reference genome annotation. The annotations of outlier SNPs and loci were summarized according to the annotated vcf and references. The position of outlier SNPs in a scaffold was visualized using trackViewer v.1.40.0 (Ou and Zhu 2019).

To determine the distribution of SNPs, all SNPs and outlier SNPs were annotated by referring to the *G. platifrons* genome annotation (Sun et al. 2017). The gff of the *G. platifrons* genome was further analyzed to determine introns and intergenic regions using AGAT v.1.2.0 (Dainat 2024). The annotation for vcf1 was conducted using snpEFF v.5.1d (Cingolani et al. 2012) with a local database built using the *G. platifrons* genome sequence and annotation file, and an interval setting of 1000 bp for both upstream and downstream regions. For vcf2, we used Python scripts to extract matched records based on annotations of Ref2, validated manually, and determined the gene type information for each SNP using snpEFF (Li 2025).

## 3 Results

### 3.1 SNP Filtering and Comparison of References

Sequencing a total of 11 multiplexed libraries for 123 individuals yielded a total of 328.4 Gb data, with an average of 2.67 Gb for each individual. Filtering resulted in 87.07%–95.58% clean reads for subsequent analysis (Table S3).

The pseudoreference Ref2 generated from this study contained 1960 UCEs with a total length of 5.36 Mbp. The total number of UCEs generated from our target capture experiment closely matched the *in silico* output of the Bivalve UCE 2k v.1 probe (1993 loci, as detailed in Table S2). Herein, we compared the results obtained from using the reference genome and pseudoreference (Table 2). The data were mapped to the pseudoreference (5.36 Mbp) and genome (1.65 Gbp), resulting in an average read depth of 66× for the pseudo‐reference and 0.46× for the genome (Table S4). The per‐base depth of each individual differed when reads were mapped to different references (Figures S1–S12), with generally higher site depths using pseudoreference (mostly < 100×, vcf2) than using the genome reference (mostly < 11×, vcf1). By mapping the sequencing data against Ref1 and Ref2, we identified 1.01 million and 439,000 raw SNPs from 1.17 million and 554,000 raw variants, respectively (Table 3). After applying stringent filtering criteria, we retained 11,807 SNPs from vcf1 and 8936 SNPs from vcf2.

**TABLE 3:** Number of SNPs retained following each step of filtering.

Putative loci after filtering steps	vcf1 (Ref1)	vcf2 (Ref2)
Raw variants	11,782,432	554,951
Raw SNPs	10,169,569	439,492
Biallelic SNPs	8,247,136	325,201
Depth > 10	7,928,211	267,375
Depth > 10 < 200	7,925,417	264,802
Exclude all missing data	56,390	42,107
Overall minor allele frequency (MAF > 0.05)	11,928	8936
Hardy–Weinberg equilibrium (*p* > 0.01)	11,807	8936

Abbreviations: Ref1, the genome of *G. platifrons*; Ref2, representative UCEs generated from all individuals.

### 3.2 Pseudoreference and SNPs Annotation

**FIGURE 2:**
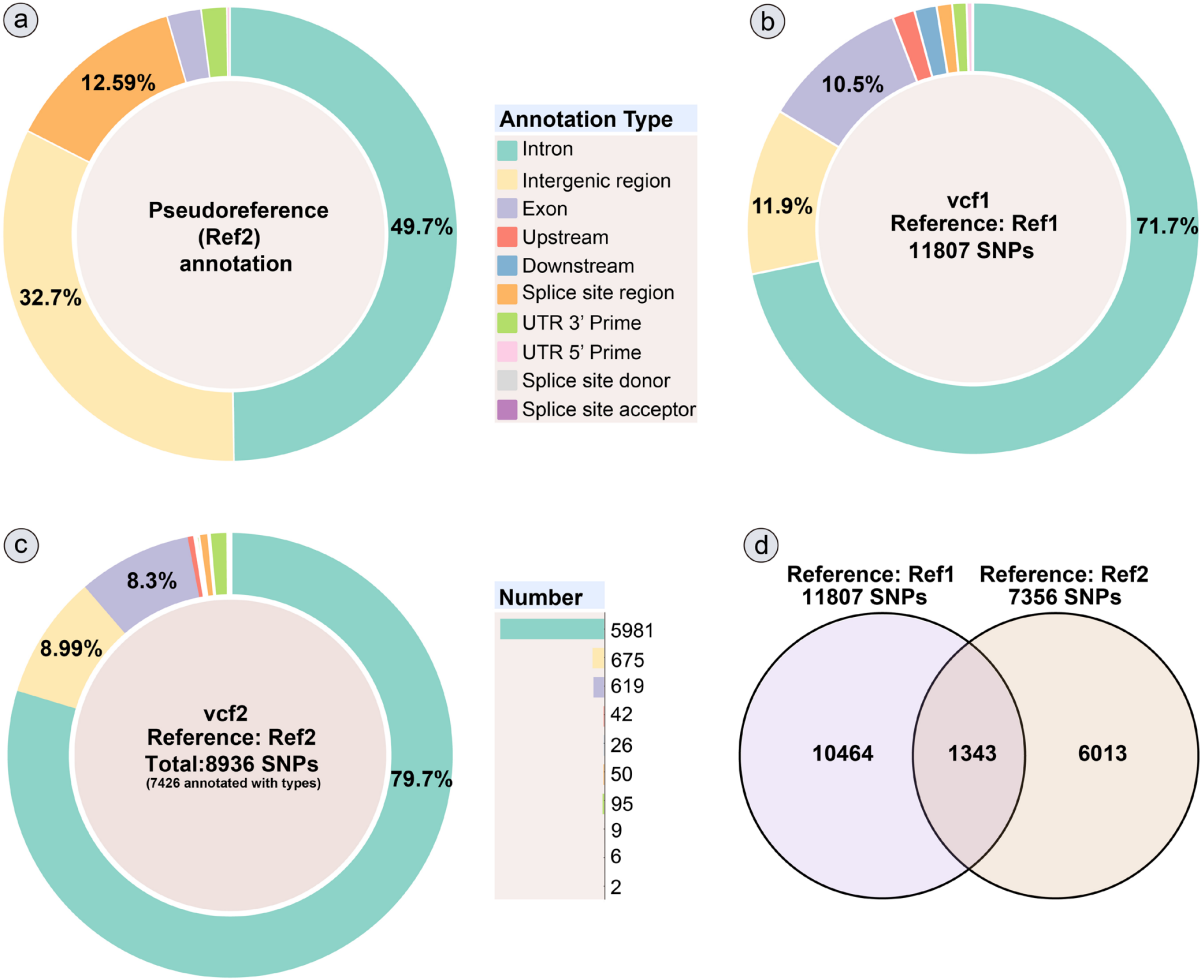
Characteristics of pseudoreference and SNP datasets. (a) composition of pseudoreference; (b) composition of SNP dataset “vcf1” (11,807 SNPs, based on genome Ref1); (c) composition of SNP dataset “vcf2” (8936 SNPs, based on pseudoreference Ref2); (d) overlap SNPs between the two SNP datasets. The annotation was obtained based on the *G. platifrons* genome. The bar chart referred to the number of annotated SNPs per annotation type.

The pseudoreference Ref2 with 1960 contigs was annotated against the genome of *G. platifrons* using BLASTn, resulting in 1927 annotated contigs with identity > 80%. The annotations showed that they were predominantly distributed within non‐coding regions (Figure 2a and Table S5). Furthermore, searching against the eggNOG online database (Table S6) yielded function annotations for 240 contigs of Ref2 (12.2% of total pseudoreference contigs).

Annotating the two SNP datasets (Figure 2b,c) derived from the two references showed that most SNPs were located in introns. For vcf1 containing 11,807 annotated SNPs (Table S7) in 1554 loci, 71.7%, 11.9%, and 10.5% of the SNPs were located in the introns (75% loci), intergenic regions (20% loci), and exons (38% loci), respectively. For vcf2 containing 8936 SNPs, although 7365 SNPs were annotated (Tables S8 and S9) using BLASTn and snpEFF, among them, the annotated SNPs were further split into different types in 1134 loci, and the distribution of the SNPs (Figure 2c) was similar to vcf1, with 79.7%, 8.99%, and 8.3% located in the introns (81% of loci), intergenic regions (15% of loci), and exons (24% of loci), respectively. Among the annotated SNPs, 1343 SNPs occurred in both vcf1 and vcf2, with 92.6% located in introns (Figure 2d, Table S10).

### 3.3 Genetic Characteristics and Differentiation

**FIGURE 3:**
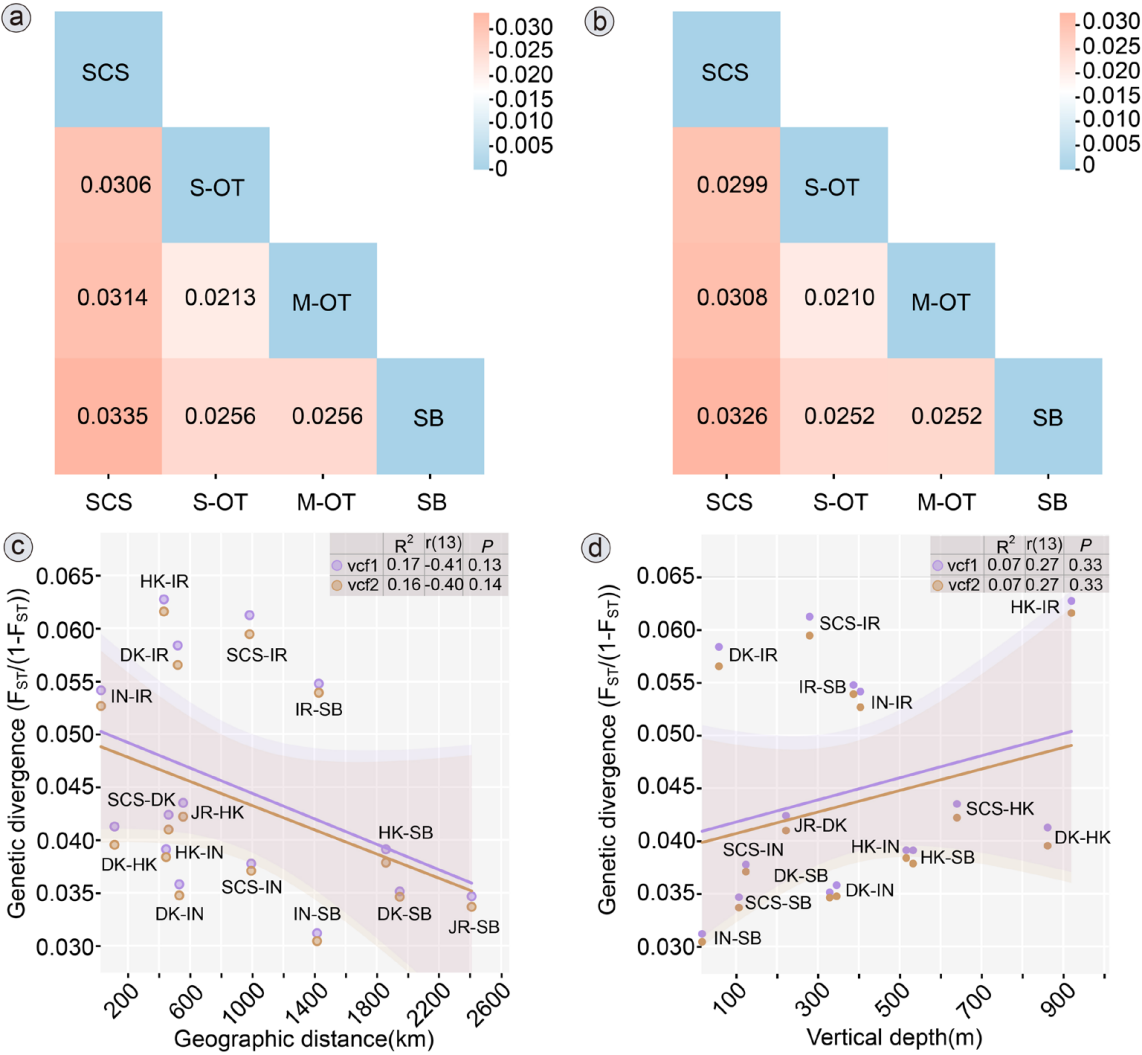
Genetic divergence within *Gigantidas platifrons*. (a and b) Summary information of *F*_ST_ between groups based on four groups, based on 11,807 SNPs (vcf1, based on genome Ref1) and 8936 SNPs (vcf2, based on pseudoreference Ref2). M‐OT, Middle Okinawa Trough (IN and IR); SB, Sagami Bay (OH); SCS, South China Sea (JR); S‐OT, Southern Okinawa Trough (DK and HK). (c and d) geographic divergence among populations based on 11,807 SNPs (vcf1) and 8936 SNPs (vcf2).

An analysis of population genetic parameters revealed similar nucleotide diversity and heterozygosity among the populations (Table 4). The range of observed heterozygosity values for each population (0.2804–0.3095) calculated in this study using UCE‐based SNPs was higher than that in a previous study (0.1480–0.1635) calculated using 2b‐RAD‐based SNPs (Xu et al. 2018). The nucleotide diversity of each group ranged from 0.1728 to 0.1759, similar to previous results (0.1594 to 0.1710, Xu et al. 2018). The genetic diversity statistics (*π* = ~0.006) estimated with invariant sites (vcf3, Table S11) were similar to those reported by Xu et al. (Xu et al. 2018; *π* = ~ 0.007). Pairwise *F*_ST_ (Figure 3a,b) between regions ranged from 0.0212 to 0.0335 for Ref1 (genome reference) and from 0.0210 to 0.0325 for Ref2 (pseudoreference), higher than in Xu et al. (2018), which ranged from −0.0010 to 0.0206. Even for the thinned datasets (vcf3 and vcf4), pairwise *F*_ST_ between regions was comparable (0.0217 to 0.0467, Table S11). No significant correlation was observed between population divergence and geographic distance (Figure 3c) or between population divergence and water depth (Figure 3d).

**TABLE 4:** Summary of genetic statistics of each local population based on 11,807 SNPs (vcf1).

Habitat	Group	*N*	*H*_obs_	*H*_exp_	*π*
Seep	JR (2018)	10	0.3110	0.3070	0.1728
Seep	JR (2013)	24	0.2830	0.2804	0.1738
Seep	OH	23	0.2859	0.2844	0.1751
Vent	DK	18	0.2861	0.2842	0.1735
Vent	HK	16	0.2904	0.2882	0.1749
Vent	IR	10	0.3107	0.3095	0.1759
Vent	IN	22	0.2877	0.2852	0.1751

Abbreviations: *π*, nucleotide diversity; DK, Dai‐yon Yonaguni; *H*_exp_, expected heterozygosity; HK, Hatoma Knoll; *H*_obs_, observed heterozygosity; IN, Iheya North.; IR, Iheya Ridge; JR, Jiaolong Ridge; *N*, number of individuals; OH, Off Hatsushima.

### 3.4 Population Genetic Structure and Dynamics

**FIGURE 4:**
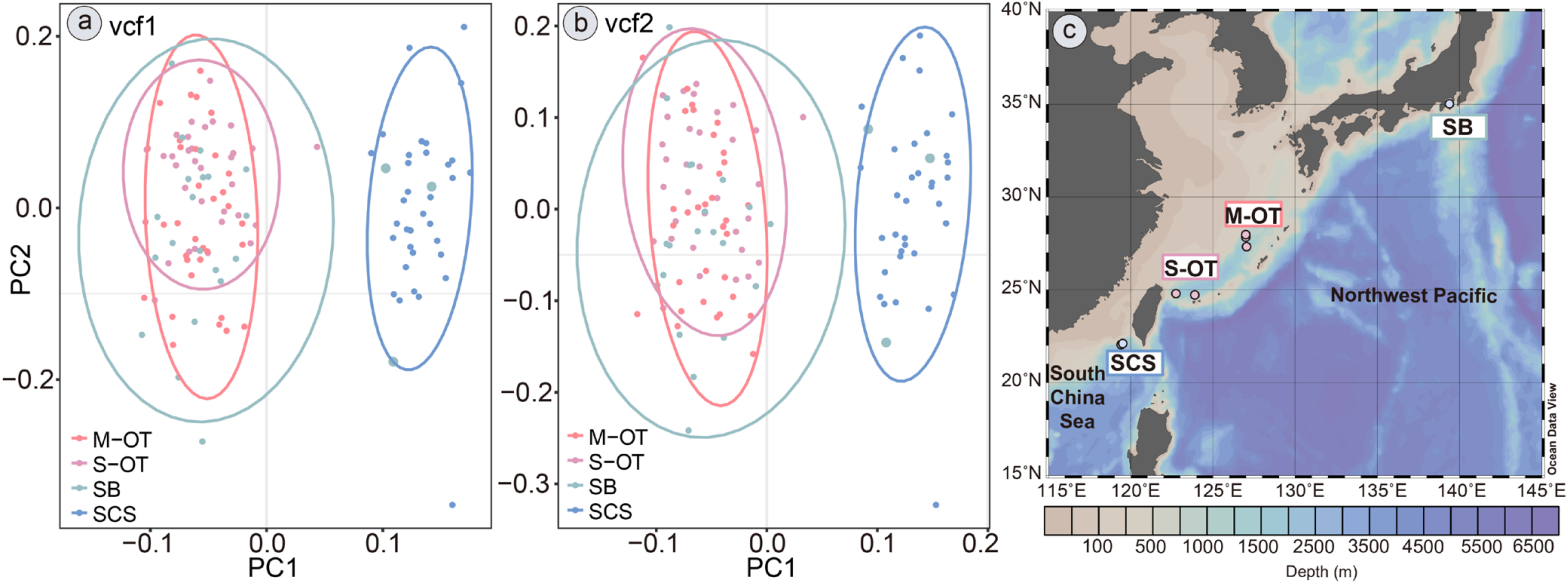
Clustering results of *G. platifrons*. (a and b) PCA results based on 11,807 SNPs (vcf1, based on genome Ref1) and 8936 SNPs (vcf2, based on pseudoreference Ref2); (c) distributions of four main lineages. M‐OT, Middle Okinawa Trough (IN and IR); SB, Sagami Bay (OH); SCS, South China Sea (JR); S‐OT, Southern Okinawa Trough (DK and HK).

Admixture analysis (Figure 1b) based on 11,807 SNPs (vcf1) and 8936 SNPs (vcf2) with the unbiased search of genetic similarities among individuals detected two distinct genetic groups (*K* = 2) across the four geographic regions—the South China Sea (SCS) group and the Okinawa Trough‐Sagami Bay (OT‐SB) group, congruent with the results of PCA and DAPC analyses (Figures 4, S13 and S14). The two datasets (vcf1 from Ref1 and vcf2 from Ref2) produced very similar clustering results (Figure 4a,b). These results again illustrated the high concordance with the 2b‐RAD approach, which detected the same two genetic groups (Xu et al. 2018), regardless of whether SNP datasets were generated by mapping UCEs to the genome or a pseudoreference (representative UCEs from all individuals).

**FIGURE 5:**
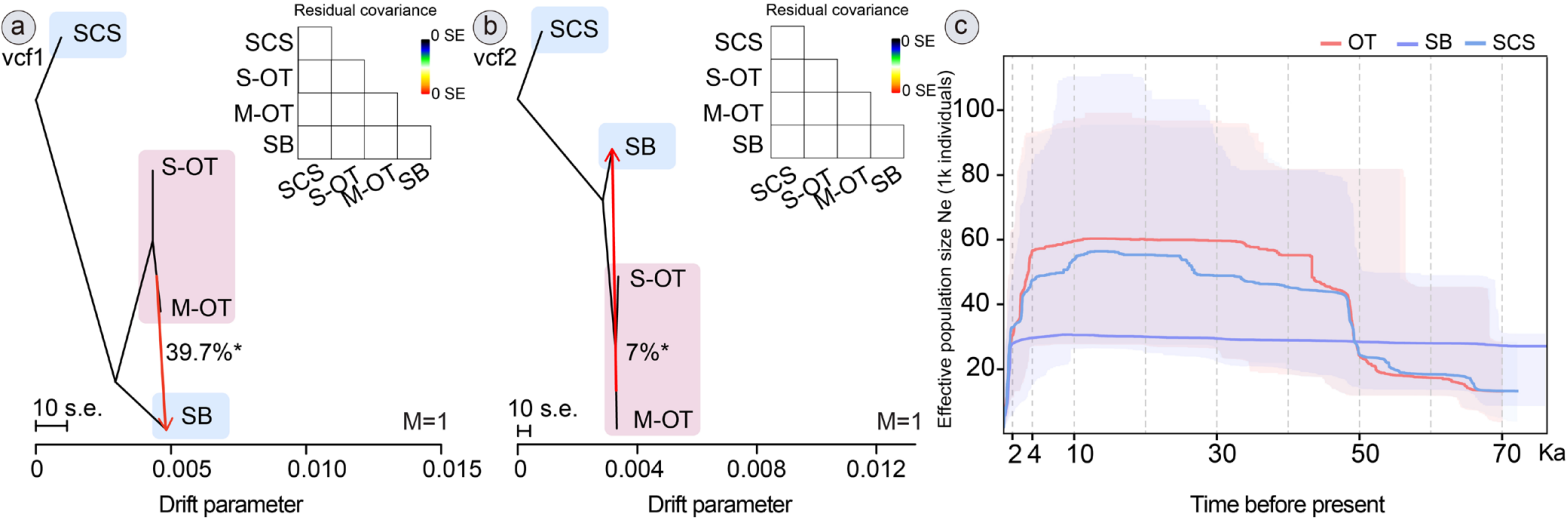
Migration events estimated by TreeMix using 11,807 SNPs (a) and 8937 SNPs (b), and demographic history illustrated using 8937 SNPs (c). M‐OT, Middle Okinawa Trough (IN and IR); OT, S‐OT and M‐OT; SB, Sagami Bay (OH); SCS, South China Sea (JR); S‐OT, Southern Okinawa Trough (DK and HK).

To detect the migration history of *G. platifrons*, we defined the tree (Figure S15) for SCS, SB, S‐OT, and M‐OT groups corresponding to geographic regions. TreeMix analysis detected a migration event (Figure 5a,b) among the four geographic groups. OptM analysis (Figures S16 and S17) confirmed the best migration edge Δm = 1 for both SNP datasets. The TreeMix trees based on the two SNP datasets both detected a significant gene flow (*p* < 0.01) from the Okinawa Trough to Sagami Bay (Figure 5a,b) with migration weights of 39.7% (vcf1) and 7% (vcf2). The migration direction from the Okinawa Trough to the Sagami Bay is congruent with Xu et al. (2018) using SNPs generated from 2b‐RAD sequencing data.

For populations from three regions, SCS, OT, and SB, we applied StairwayPlot2 to estimate their recent demographic dynamics (Figure 5c). The SCS and OT groups presented an increase, followed by a long‐term plateau, and then a dramatic decrease over the last 4000 years. The effective population size of the SB group showed a long‐period plateau and was lower than that of the SCS and OT groups until the last 2000 years, followed by a similar shrinkage tendency as the SCS and OT groups.

### 3.5 Genetic Signal of Contrasting Selection Across Regions

**FIGURE 6:**
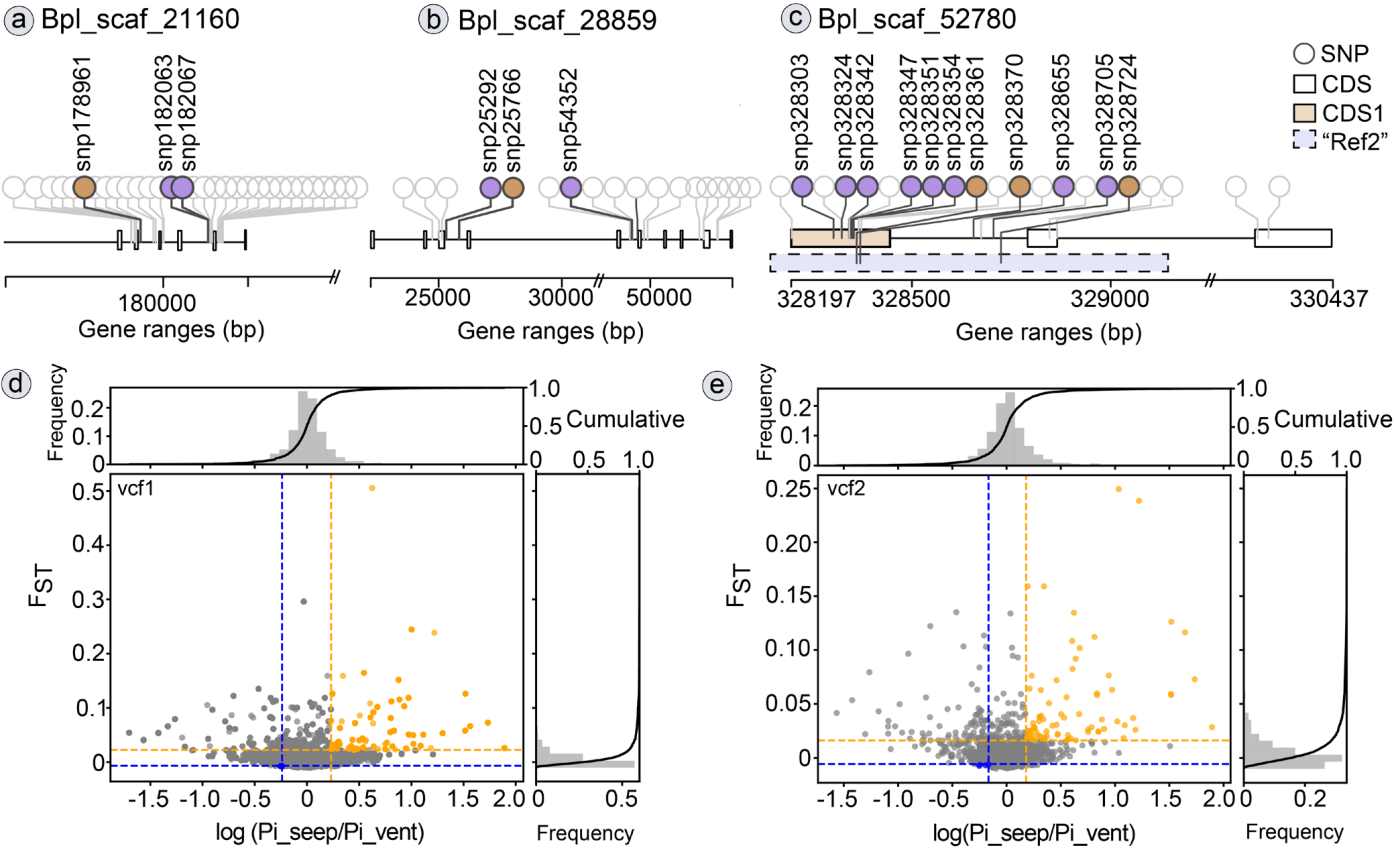
Distribution of outlier SNPs in a respective range in three scaffolds (a–c) of *G. platifrons* and selective analysis (10‐kb windows sliding in 2‐kb steps) for seep groups and vent groups using vcf1 (d) and vcf2 (e). Circle lollipops, SNPs from “vcf1” and “vcf2”; lollipops in blue color, outlier SNPs from “vcf1”; lollipops in pink color, outlier SNPs from “vcf2”; label of SNP, position of outlier SNP; block in axis, CDS; CDS1, the first CDS of Bpl_scaf_52780–3.4; “Ref2,” UCE v2‐3349 mapped against CDS1. Pi_seep and Pi_vent, nucleotide diversity of the seep and vent groups; yellow circles, selected data in the high tail of log(Pi_seep/Pi_vent) and *F*_*ST*_; blue circles, selected data in the low tail of log(Pi_seep/Pi_vent) and *F*_*ST*_.

To detect any potential differentiation between the four regions, we first identified outlier SNPs using BayeScan (Table S12), which recognized 23 outlier SNPs from vcf1 (Figure S18) and 10 outlier SNPs from vcf2 (Figure S19). Among these 33 outlier SNPs, 17 SNPs were located on three shared scaffolds (Figure 6a–c, Table S12), mostly belonging to non‐coding regions (Figures 2 and 6a–c). We also examined the selective signal in the mussel genome (Figures S20 and S21, Tables S13 and S14) based on *F*_ST_ and π ratio or log‐transformed π ratio (Figure 6d–e, Table S15) to some functional regions that are closely relevant to the impact of the Luzon Strait as a geographic barrier (SCS vs. OT‐SB) and seep‐vent habitat (SCS‐SB vs. OT).

Based on the π ratio and *F*_ST_, a total of 112 candidate genes in 41 scaffolds (Table S13) were identified for the SCS group compared to OT‐SB. Comparing the vent group (OT), 127 candidate genes in 69 scaffolds (Table S14) were yielded for the seep group (SB and SCS). Two variant datasets shared 16 differentiated genes (Table S13) and could contribute to the differentiation of SCS individuals (Jiaolong Ridge) from the northern individuals. Comparing across habitats, we identified 12 candidate genes in both VCF datasets (Table S14), which may reflect the diversification of seep regions. Notably, eight areas with selective signals, including six missense and two synonymous variants, were detected in the first coding region of a gene associated with detoxification (Bpl_scaf_52780‐3.4, melanotransferrin‐like isoform X1, Figure 6c, Tables S12 and S14). Our log‐transformed analysis identified a total of 92 and 111 candidate genes for the SCS and seep groups, respectively, using two variant datasets (Table S15), encompassing 86%–97% of the selected regions in the high tail of the distributions (Figures 6d and S22). Although < 10 shared candidate genes were detected in the two datasets, nine areas with selective signals were detected in the intron region of Bpl_scaf_12351‐1.2 (aryl hydrocarbon receptor, AHR), which is involved in cellular responses to stress, pollutant metabolism, and environmental signals (Table S15). Notably, signals were detected in Bpl_scaf_52012‐2.9 (pre‐mRNA processing splicing factor 8, PRPF8, the catalytic center of the spliceosome) and Bpl_scaf_48578‐1.5 (anion exchange 2‐like isoform X1, AE2, and osmoregulation), only from vcf2 and obtained using pseudoreference Ref2 (Table S15).

## 4 Discussion

### 4.1 Pseudoreference Composition and Its Potential in Marine Invertebrate Study

UCE target capture sequencing is a powerful evolutionary tool for studying marine invertebrates, particularly due to its ability to generate genome‐wide single‐nucleotide polymorphisms (SNPs) from low‐quality or degraded DNA, a common challenge in deep‐sea or historically preserved samples (Faircloth et al. 2012; Byerly et al. 2024). UCE‐captured sequencing enables robust SNP calling without a high‐quality reference genome, facilitating evolutionary studies of population structure and adaptation in non‐model marine species (Chan et al. 2020; Glon et al. 2021). By contrast, RAD‐Seq with a higher requirement for DNA quality is limited by uneven coverage and paralog confusion in complex marine genomes (Hohenlohe et al. 2011; Vendrami et al. 2017). To validate the efficacy of UCE‐captured pseudoreference, we provided a detailed workflow for constructing a UCE‐based pseudoreference (Ref2) and examined its composition (Tables 3 and S5, Figure 2a). The proportion of coding regions (~2.5%) in Ref2 is lower than the range observed in previous records (9.3%–82.3%) in UCE target capture studies for both vertebrates and invertebrates (Van Dam et al. 2021; Snetkova et al. 2022). This discrepancy may be due to the influence of genomic/transcriptomic assemblies used in their probe design (Van Dam et al. 2021). Most UCEs are found predominantly located in the intronic and intergenic regions, and their functions may be critical for the fitness and development of organisms through gene regulation (Kern et al. 2015; McCole et al. 2018; Cummins et al. 2024). From our 1960 Ref2 contigs, we annotated 243 UCE coding assemblies via eggNOG searches, including some conserved genes in invertebrates (Table S6), for instance, *ADK* (larvae settlement, He et al. 2021), *LRIG3* (osmotic stress response, Zhao et al. 2012), *Keap1* (inhibitor of detoxification of reactive oxygen species, Wang et al. 2021), *MAPK10* (response to environmental stress, Zhang et al. 2023), *NudC* and *KCNA2* (conserved in eukaryotes, Hoegg and Meyer 2007; Zheng et al. 2011), among others.

The technical validity of our study was demonstrated by the sequencing depth for *G. platifrons* populations using pseudoreference (66× clean reads on average), higher than those in several published UCE studies (Lim and Braun 2016; Winker et al. 2018; Erickson et al. 2021; Stiller et al. 2021). To validate the effectiveness of UCE‐based SNPs, we identified 11,807 (vcf1) and 8936 (vcf2) biallelic SNPs (Table 3) from UCE target‐enriched sequencing data after mapping and filtration of low‐quality variants. Our outputs were comparable with previous deep‐sea population genomic studies (1145–32,452 biallelic SNPs) that applied transcriptome sequencing (Xiao et al. 2020), RAD‐Seq (Cheng et al. 2020; DeLeo et al. 2022), 2b‐RAD sequencing (Xu et al. 2018; Zhang et al. 2024), GBS‐seq (Genotype by Sequencing, Everett et al. 2016; Xu et al. 2021, 2024; van der Reis et al. 2022) to deep‐sea coral, lobsters, gastropods, and mussels. Nevertheless, the overlap between the two SNP datasets was low, which might be due to the use of different references, as the genome assembly relied on a single individual (Sun et al. 2017), whereas the pseudoreference incorporated contigs from 123 individuals and might therefore include variants that were absent from the results obtained by mapping to the genome. Utilizing two reference choices, we further explored the efficiency of UCE‐based SNPs in unraveling the genetic diversity of populations. We observed comparable yields of SNP variants and consistent population structure (Table 4, Figures 1b, 3, and 4), indicating the pseudoreference is also a robust tool for downstream analysis of marine invertebrate population studies.

### 4.2 Population Diversity and Connectivity Unraveled by UCE‐Based SNPs

UCE‐based SNPs provide high‐resolution data to address evolutionary questions about population connectivity and diversity in marine systems on a fine scale (Stiller et al. 2021). Our UCE‐based analyses revealed pronounced genetic subdivision between SCS and other Pacific populations (Table 4, Figure 3), congruent with 2b‐RAD findings (Xu et al. 2018) but undetected by mitochondrial genes in Xu et al. (2018) and earlier studies (Kyuno et al. 2009; Miyazaki et al. 2013; Shen et al. 2016). Our variant‐only estimates are slightly higher than in Xu et al. (2017). To ensure comparability, we generated new datasets to estimate genetic diversity with invariant sites (Table S11), which yielded slightly higher values than those obtained from 2b‐RAD data, with heterozygosity of 0.010–0.012, π values of 0.00605–0.00646, and *F*_*ST*_ values of 0.028–0.047. The lower values in 2b‐RAD likely reflect allele dropout (Davey et al. 2012; Cariou et al. 2016) compared to UCE target capture's more uniform coverage.

Differentiation between groups from the SCS and OT regions (*F*_*ST*_, 0.0357–0.0577, Table S16) was also observed for the abyssochrysoidean snail *Provanna subglabra* (0.109, misidentified as *P. glabra* in Zhang et al. 2024), much lower divergence than the deep‐sea squat lobster *Shinkaia crosnieri* (0.2062–0.2301, Xu et al. 2024) and the limpet *Bathyacmaea nipponica* (0.3415–0.5280, Xu et al. 2021). The two gastropod species, *B. nipponica* and *P. subglabra* , exhibited significant differences in their levels of differentiation, which may be due to limited sampling efforts (only 30 *P. subglabra* individuals collected from three localities), as indicated by previous studies on squat lobsters (Cheng et al. 2020; Xiao et al. 2020; Xu et al. 2024), or it could reflect differences in their life history traits and dispersal capabilities. Previous studies on *B. nipponica* and *G. platifrons* applied similar sampling scales, but the degree of genetic differentiation between regions in *B. nipponica* limpets (0.0934–0.6137) was much higher than in *G. platifrons* mussels (Xu et al. 2018 and this study). This likely reflects their differences in divergence time, historic population size changes, life history traits (lecithotrophic vs. planktotrophic, generation time), and dispersal capability (middle and deep water vs. upper ocean layer, Arellano et al. 2014; Ponder et al. 2020; Xu et al. 2021).

The population structures of deep‐sea fauna in the Pacific Ocean have been a prominent topic over the past two decades, providing substantial data for understanding their genetic connectivity (Ritchie et al. 2016; Taylor and Roterman 2017; Xu et al. 2021; Tran et al. 2022). Our genetic structure analysis, using two UCE‐based SNP datasets (Figure 5), aligns with early investigations of Western Pacific deep‐sea benthos from chemosynthetic ecosystems (Xu et al. 2018, 2021, 2024; Cheng et al. 2020; Zhang et al. 2024), supporting the notion that the SCS and OT populations are genetically distant. Regarding genetic divergence among geographic sites (Figures 3 and 5), it appears that habitat types (seep or vent) and geographic differences may not be the major factors driving diversification in *G. platifrons*. The Luzon Strait emerges as a significant barrier, limiting connectivity by restricting pelagic larval dispersal between the SCS and northern regions due to its topography and reduced water volumes (Andres et al. 2015; Xu et al. 2024). This constraint likely contributes to the genetic subdivision of the SCS population compared to others.

### 4.3 Adaptation of Deep‐Sea Mussels and Their Conservation Implications

Accumulation of genetic divergence over time may lead to population divergence. To detect differentiation, the features of variants are essential for unraveling the adaptation of deep‐sea organisms in response to environmental factors, such as geographic barriers, depth, and sulfide exposure (Xu et al. 2017; Gaither et al. 2018). UCEs provide uniform coverage and high‐resolution SNP data, which are critical for identifying selective signals in non‐model marine species. In this study, we compared two variant datasets and examined selective signals between SCS and other populations, as well as between seep and vent individuals. We identified some candidate genes of SCS individuals as potential genes associated with differentiation in the Jiaolong Ridge. These genes were involved in pathways related to detoxification (e.g., phenylalanine tRNA ligase alpha subunit‐like, Zhang et al. 2020) and hypoxia response (e.g., tubulin epsilon chain‐like, Bpl_scaf_22826–0.18, Luo et al. 2024).

Intriguingly, eight outlier SNPs were found using BayeScan, located within the first CDS of gene Bpl_scaf_522780‐3.4 (comprising six missense and two synonymous variants), despite the majority of outlier SNPs occurring in non‐coding regions (Figure 2, Table S10). Selective signals were detected in the melanotransferrin‐like gene (Bpl_scaf_522780‐3.4) between seep and vent individuals (Figure 2, Table S13), which is implicated in H_2_S detoxification pathways in the hydrocarbon seep conoidean snail *Phymorphynchus buccinoides* (Liu et al. 2023). Unlike synonymous variants, missense mutations may impact the functional and structural stability of genes (Bowman et al. 2018; Zhang et al. 2012). Therefore, the presence of six missense outlier SNPs (vcf1) within the melanotransferrin‐like gene may imply that *G. platifrons* is undergoing selection for detoxification, possibly induced by different environmental conditions. Selection signals (Table S14) in PRPF8 (Bpl_scaf_52012‐2.9) and AE2 (Bpl_scaf_48578‐1.5) may also enhance the efficiency of stress tolerance (Ma et al. 2025) and osmoregulation (Grosell et al. 2009). Moreover, signals in AHR (Bpl_scaf_12351‐1.2) were detected between seeps and vents, as well as between SCS and other North Pacific sites (Table S14), suggesting that pollutant detoxification (Châtel et al. 2012; Zeng et al. 2024) represents a common adaptive challenge for seep individuals. These signals may influence the habitat preference and enhance the dispersal capacity of seep individuals, leading to limited genetic admixture primarily between SCS and SB regions (Figure 1b), also indicated by the observation of three individuals from Off Hatsushima (SB) grouping with the JR population (Figures 4 and S23). The same genetic admixture phenomenon between SCS and SB has been reported by 2b‐RAD results of Xu et al. (2018), requiring validation with more specimens from Off Hatsushima (SB) in the future.

Given the endemic diversity of the SCS seep communities (He et al. 2023; Li et al. 2023; Zhao et al. 2020), the limited connectivity of these communities with other regions underscores their critical conservation value, especially amid emerging gas/methane hydrate exploration and production in the Northern SCS (Lu et al. 2023; Zhang, Liang, et al. 2021). Notably, test mining of massive sulfides has already begun in the OT (Okamato et al. 2019). Washburn et al. (2023) observed the enduring impact of even small‐scale operations on macrobenthos, lasting over 3 years, and emphasized the critical need for robust scientific evidence to develop effective conservation strategies. Vent sites in the OT have been posited as sources of gene flow to SCS (lobster *S. crosnieri* ; Xu et al. 2024) and SB (mussel *G. platifrons*, Figure 5a,b). Moreover, our UCE‐based SNPs estimated declining population sizes for three mussel groups (Figure 5c), suggesting a potential bottleneck. Although extensive mussel beds were discovered from in situ (Zhao et al. 2020; Watanabe et al. 2010), our estimation of population size decrease may reflect their sensitivity to fluid activity changes over a long period, such as reduced fluid supply in the Jiaolong Ridge over the past 2000 years (Feng and Chen 2015). Precise paleoenvironmental models and expanded genetic data on flag species are needed to assess the resilience of deep‐sea ecosystems in the Northwest Pacific. Overall, our confirmation of genetic divergence in *G. platifrons* using new UCE‐based SNPs reiterates the importance of regional conservation management in Western Pacific chemosynthetic ecosystems, particularly highlighting the biological significance of OT vents, as evidenced by the aforementioned biogeography studies of macrofauna.

### 4.4 Strength of UCE Target Capture Sequencing in Marine Conservation

Population genomics has become pivotal in conservation and management by deciphering both neutral and selective variations, thereby elucidating the underlying forces that shape the genetic diversity and connectivity among populations (Crawford and Oleksiak 2016; Oleksiak and Rajora 2020; Yue et al. 2020; Mao et al. 2024). Reduced representation sequencing (i.e., RAD‐Seq and target capture sequencing) has emerged as a potent and cost‐effective strategy that enables the efficient evaluation of genetic diversity, population structure, and adaptive genetic variation in marine non‐model species (Fu et al. 2016; Glon et al. 2021). To date, several published studies have used genomic approaches to identify genetic groups of deep‐sea benthos (e.g., Xiao et al. 2020; Xu et al. 2021, 2024; van der Reis et al. 2022), including ones that applied RAD‐Seq data (e.g., Xu et al. 2018; Cheng et al. 2020; Zhang et al. 2024).

The pooling strategy we applied (10 or 12 individuals per pool) results in a wet‐lab expense of UCE capture sequencing of approximately US$80 per individual (Table S17), with an average data yield of 2.67 Gb (8.9 million reads, 66× based on pseudoreference). In comparison to non‐selective sequencing approaches (Li et al. 2018; Qi et al. 2021), such as resequencing (US$244 per individual, 30 Gb data with 100 million reads, ~18× based on genome size of *G. platifrons*), UCE target capture sequencing can achieve comparable depth (> 20 ×, Lim and Braun 2016; Vinciguerra et al. 2019; this study) at 50% lower cost, with per‐individual expenses (US$80) similar to 2b‐RAD sequencing (US$67, 6 million reads). While RAD seq is marginally cheaper, UCE sequencing offers greater robustness by avoiding allele dropout and restriction site bias, and has wide‐scale applicability across taxonomic groups, particularly in degraded marine samples. Notably, computational demands for UCE sequencing data are relatively low and easy to handle due to low sequencing volume and mature analysis pipelines (Faircloth 2016; Brennan et al. 2024). Despite lower genomic coverage than whole genome resequencing, our study corroborates existing evidence (Erickson et al. 2021; Stiller et al. 2021) that UCE sequencing provides sufficient resolution to delimit conservation units for management and address critical evolutionary questions regarding population structure, gene flow, and adaptation in deep‐sea ecosystems.

## 5 Conclusion

UCE target capture sequencing offers a powerful approach for studying non‐model marine species, particularly in challenging deep‐sea environments where sample quality is often compromised. To demonstrate this, we successfully generated a representative pseudoreference from UCEs captured from all assemblies using the probe set “Bivalve UCE 2k v.1” and performed population genetic analyses for the deep‐sea mussel *Gigantidas platifrons* as a case study. This study demonstrated the comparable effectiveness of the pseudoreference and the whole genome for UCE mapping, revealing two cryptic, semi‐isolated *G. platifrons* lineages in the Northwest Pacific Ocean: the SCS and OT‐SB lineages. These findings provide proof‐of‐concept that even without a reference genome, UCE‐captured SNPs offer valuable insights into population genetics analyses by detecting connectivity spanning diverse environments, whether in the deep sea (this study; Gordon 2023) or shallow waters (Stiller et al. 2021). Moreover, “Bivalve UCE 2k v.1” UCE probes specifically designed for Bivalvia demonstrated robustness and versatility across diverse evolutionary investigations from intraspecific biogeographic studies to high‐level phylogenomics (Li et al. 2025). Unlike the 2b‐RAD approach discussed in previous comparison studies (Erickson et al. 2021), the UCE target‐enriched technique has advantages on a broader application scale, with more uniform coverage and reduced paralog errors. This universal UCE marker system is poised to significantly advance marine biodiversity conservation by elucidating evolutionary relationships and informing evidence‐based management strategies in marine ecosystems.

## Funding

This work was supported by the Collaborative Research Fund (grant C2013‐22GF) and the General Research Fund (grants 12101021, 12102222, and 16309324) from the Research Grants Council of Hong Kong.

## Conflicts of Interest

The authors declare no conflicts of interest.

## Supporting information


**Figure S1:** Summary of site depth for JR population based on vcf1 (1× − 30×).
**Figure S2:** Summary of site depth for SB population based on vcf1 (1× − 30×).
**Figure S3:** Summary of site depth for DK population based on vcf1 (1× − 30×).
**Figure S4:** Summary of site depth for HK population based on vcf1 (1× − 30×).
**Figure S5:** Summary of site depth for IR population based on vcf1 (1× − 30×).
**Figure S6:** Summary of site depth for IN population based on vcf1 (1× − 30×).
**Figure S7:** Summary of site depth for JR population based on vcf2 (1× − 200×).
**Figure S8:** Summary of site depth for SB population based on vcf2 (1× − 200×).
**Figure S9:** Summary of site depth for DK population based on vcf2 (1× − 200×).
**Figure S10:** Summary of site depth for HK population based on vcf2 (1× − 200×).
**Figure S11:** Summary of site depth for IR population based on vcf2 (1× − 200×).
**Figure S12:** Summary of site depth for IN population based on vcf2 (1× − 200×).
**Figure S13:** Admixture results (K = 2–4) for vcf1 (a) and vcf2 (b).
**Figure S14:** DAPC result of vcf1 and vcf2 with K = 2 and K = 4.
**Figure S15:** Migration analysis by TreeMix using vcf1 (a) and vcf2 (b) with “m = 0” setting.
**Figure S16:** Optimal migration edge analysis for vcf1 using OptM.
**Figure S17:** Optimal migration edge analysis for vcf2 using OptM.
**Figure S18:** BayeScan results using vcf1 dataset. The number near black circle, site in vcf1 dataset.
**Figure S19:** BayeScan results using vcf2 dataset. The number near black circle, site in vcf2 dataset.
**Figure S20:** *F*_*ST*_ and π ratio analysis for individuals from seep and vent using vcf1 (a) and vcf2 (b). Pi_seep and Pi_vent, nucleotide diversity of the seep groups and the vent groups; Red data points located to the right of vertical dashed lines and above the horizontal dashed line, F_ST_ above 0.05 and in the top 10% right tail of the empirical nucleotide diversity distribution (Pi = 1.48 for vcf1 and Pi = 1.28 for vcf2).
**Figure S21:** *F*_*ST*_ and π ratio analysis for individuals from SCS and OT‐SB using vcf1 (a) and vcf2 (b). Pi_seep and Pi_vent, nucleotide diversity of the seep groups and the vent groups; Red data points located to the right of vertical dashed lines and above the horizontal dashed line, F_ST_ above 0.05 and in the top 10% right tail of the empirical nucleotide diversity distribution (Pi = 1.48 for vcf1 and Pi = 1.28 for vcf2).
**Figure S22:** F_ST_ and log‐transformed π ratio analysis for individuals from seeps and vents using vcf1 (a) and vcf2 (b).
**Figure S23:** PCA analysis for four linages (SCS, S‐OT, M‐OT, and SB) using vcf3 (2064 SNPs, mapped with genome reference) and vcf4 (1811 SNPs, mapped with pseudoreference). Details of vcf3 and vcf4 in Table S16.

**Table S1:** Sampling information of Gigantidas platifrons.
**Table S2:** Information for UCE capture for Gigantidas platfrons (Bivalve UCE 2k v.1).
**Table S3:** Sequencing information.
**Table S4:** Coverage and depth information.
**Table S5:** Annotation of Pseudoreference (Ref2).
**Table S6:** Functional annotation of Pseudoreference (Ref2).
**Table S7:** Annotation for 11,807 SNPs (vcf1).
**Table S8:** Annotation for 7356 SNPs (vcf2) by BLASTn.
**Table S9:** Annotation for 7356 SNPs (vcf2) by snpEFF.
**Table S10:** Annotation for 1343 overlapped SNPs between the two SNP datasets.
**Table S11:** Genetic diversity statistics using filtered vcf files (sliding window 10,000 bp) by Pixy and vcftools.
**Table S11:** Annotations of outlier SNPs between four regions using Bayescan.
**Table S12:** Description for selective signals located genes using (SCS vs. OT‐SB, pi ratio).
**Table S13:** Description for selective signals located genes (Seep vs. Vent, pi ratio).
**Table S14:** Description for selective signals located genes (log pi ratio).
**Table S16:** F_ST_ results of six populations by using two variant datasets (a. vcf1, b. vcf2).
**Table S17:** Wet‐lab expenses in China in 2024.

## Data Availability

Raw sequence reads are available in NCBI's SRA database under Bioproject PRJNA1132735. Genotype data and Python scripts for SNP annotation are deposited in FigShare (Li 2025).

## References

[eva70195-bib-0001] Aguirre‐Liguori, J. A. , J. A. Luna‐Sánchez , J. Gasca‐Pineda , and L. E. Eguiarte . 2020. “Evaluation of the Minimum Sampling Design for Population Genomic and Microsatellite Studies: An Analysis Based on Wild Maize.” Frontiers in Genetics 11: 870. 33193568 10.3389/fgene.2020.00870 PMC7531271

[eva70195-bib-0002] Alexander, D. H. , and K. Lange . 2011. “Enhancements to the ADMIXTURE Algorithm for Individual Ancestry Estimation.” BMC Bioinformatics 12: 1–6. 21682921 10.1186/1471-2105-12-246 PMC3146885

[eva70195-bib-0003] Andres, M. , S. Jan , T. Sanford , V. Mensah , L. Centurioni , and J. Book . 2015. “Mean Structure and Variability of the Kuroshio From Northeastern Taiwan to Southwestern Japan.” Oceanography 28, no. 4: 84–95.

[eva70195-bib-0004] Arellano, S. M. , A. L. Van Gaest , S. B. Johnson , R. C. Vrijenhoek , and C. M. Young . 2014. “Larvae From Deep‐Sea Methane Seeps Disperse in Surface Waters.” Proceedings of the Royal Society B: Biological Sciences 281, no. 1786: 20133276. 10.1098/rspb.2013.3276 PMC4046398 24827437

[eva70195-bib-0005] Aristide, L. , and R. Fernández . 2023. “Genomic Insights Into Mollusk Terrestrialization: Parallel and Convergent Gene Family Expansions as Key Facilitators in Out‐Of‐The‐Sea Transitions.” Genome Biology and Evolution 15, no. 10: evad176. 37793176 10.1093/gbe/evad176 PMC10581543

[eva70195-bib-0006] Bolger, A. M. , M. Lohse , and B. Usadel . 2014. “Trimmomatic: A Flexible Trimmer for Illumina Sequence Data.” Bioinformatics 30, no. 15: 2114–2120. 24695404 10.1093/bioinformatics/btu170 PMC4103590

[eva70195-bib-0007] Bowman, L. L. , E. S. Kondrateva , M. A. Timofeyev , and L. Y. Yampolsky . 2018. “Temperature Gradient Affects Differentiation of Gene Expression and SNP Allele Frequencies in the Dominant Lake Baikal Zooplankton Species.” Molecular Ecology 27, no. 11: 2544–2559. 29691934 10.1111/mec.14704

[eva70195-bib-0008] Brennan, I. G. , S. Singhal , and Z. A. Bkhetan . 2024. “Pipesnake: Generalized Software for the Assembly and Analysis of Phylogenomic Datasets From Conserved Genomic Loci.” Bioinofrmatics 40, no. 5: btae195. 10.1093/bioinformatics/btae195 PMC11082421 38597877

[eva70195-bib-0011] BroadInstitute . 2019. “Picard Toolkit.” In GitHub Repository. Broad Institute. https://broadinstitute.github.io/picard/.

[eva70195-bib-0012] Byerly, P. A. , A. M. Kearns , A. Welch , et al. 2024. “Museum Genomics Provide Insight Into the Extinction of a Specialist North American Warbler Species.” Scientific Reports 14: 17047. 39048633 10.1038/s41598-024-67595-5 PMC11269716

[eva70195-bib-0013] Cantalapiedra, C. P. , A. Hernández‐Plaza , I. Letunic , P. Bork , and J. Huerta‐Cepas . 2021. “eggNOG‐Mapper v2: Functional Annotation, Orthology Assignments, and Domain Prediction at the Metagenomic Scale.” Molecular Biology and Evolution 38, no. 12: 5825–5829. 34597405 10.1093/molbev/msab293 PMC8662613

[eva70195-bib-0014] Cariou, M. , L. Duret , and S. Charlat . 2016. “How and How Much Does RAD‐Seq Bias Genetic Diversity Estimates?” BMC Evolutionary Biology 16, no. 1: 240. 27825303 10.1186/s12862-016-0791-0 PMC5100275

[eva70195-bib-0015] Challis, R. , S. Kumar , C. Sotero‐Caio , M. Brown , and M. L. Blaxter . 2023. “Genomes on a Tree (GoaT): A Versatile, Scalable Search Engine for Genomic and Sequencing Project Metadata Across the Eukaryotic Tree of Life.” Wellcome Open Research 8: 24. https://wellcomeopenresearch.org/articles/8‐24/v1. 36864925 10.12688/wellcomeopenres.18658.1 PMC9971660

[eva70195-bib-0016] Chan, K. O. , C. R. Hutter , P. L. Wood , L. L. Grismer , I. Das , and R. M. Brown . 2020. “Gene Flow Creates a Mirage of Cryptic Species in a Southeast Asian Spotted Stream Frog Complex.” Molecular Ecology 29, no. 20: 3970–3987. 32808335 10.1111/mec.15603

[eva70195-bib-0017] Châtel, A. , V. Faucet‐Marquis , M. Perret , et al. 2012. “Genotoxicity Assessment and Detoxification Induction in *Dreissena polymorpha* Exposed to Benzo[a]Pyrene.” Mutagenesis 27: 703–711. 22844080 10.1093/mutage/ges036

[eva70195-bib-0018] Chen, D. , E. L. Braun , M. Forthman , R. T. Kimball , and Z. Zhang . 2018. “A Simple Strategy for Recovering Ultraconserved Elements, Exons, and Introns From Low Coverage Shotgun Sequencing of Museum Specimens: Placement of the Partridge Genus *Tropicoperdix* Within the Galliformes.” Molecular Phylogenetics and Evolution 129: 304–314. 30201427 10.1016/j.ympev.2018.09.005

[eva70195-bib-0019] Cheng, J. , M. Hui , Y. Li , and Z. Sha . 2020. “Genomic Evidence of Population Genetic Differentiation in Deep‐Sea Squat Lobster *Shinkaia crosnieri* (Crustacea: Decapoda: Anomura) From Northwestern Pacific Hydrothermal Vent and Cold Seep.” Deep Sea Research Part I: Oceanographic Research Papers 156: 103188.

[eva70195-bib-0020] Cingolani, P. , A. Platts , L. L. Wang , et al. 2012. “A Program for Annotating and Predicting the Effects of Single Nucleotide Polymorphisms, SnpEff.” Fly 6, no. 2: 80–92. 22728672 10.4161/fly.19695 PMC3679285

[eva70195-bib-0021] Crawford, D. L. , and M. F. Oleksiak . 2016. “Ecological Population Genomics in the Marine Environment.” Briefings in Functional Genomics 15, no. 5: 342–351. 27044302 10.1093/bfgp/elw008

[eva70195-bib-0022] Cummins, M. , C. Watson , R. J. Edwards , and J. S. Mattick . 2024. “The Evolution of Ultraconserved Elements in Vertebrates.” Molecular Biology and Evolution 41, no. 7: msae146. 39058500 10.1093/molbev/msae146 PMC11276968

[eva70195-bib-0023] Dainat, J. 2024. “AGAT: Another Gff Analysis Toolkit to Handle Annotations in Any GTF/GFF format (Version v0.8.0).” *Zenodo*. 10.5281/zenodo.3552717.

[eva70195-bib-0139] Danecek, P. , A. Auton , G. Abecasis , et al. 2011. “The Variant Call Format and VCFtools.” Bioinformatics 27, no. 15: 2156–2158. 10.1093/bioinformatics/btr330. 21653522 PMC3137218

[eva70195-bib-0024] Danecek, P. , J. K. Bonfield , J. Liddle , et al. 2021. “Twelve Years of SAMtools and BCFtools.” GigaScience 10, no. 2: 1–4. 10.1093/gigascience/giab008 PMC7931819 33590861

[eva70195-bib-0025] Davey, J. W. , T. Cezard , P. Fuentes‐Utrilla , et al. 2012. “Special Features of RAD Sequencing Data: Implications for Genotyping.” Molecular Ecology 22, no. 11: 3151–3164. 23110438 10.1111/mec.12084 PMC3712469

[eva70195-bib-0026] DeLeo, D. M. , C. L. Morrison , M. Sei , V. Salamone , A. W. J. Demopoulos , and A. M. Quattrini . 2022. “Genetic Diversity and Connectivity of Chemosynthetic Cold Seep Mussels From the U.S. Atlantic Margin.” BMC Ecology and Evolution 22, no. 1: 76. 35715723 10.1186/s12862-022-02027-4 PMC9204967

[eva70195-bib-0027] Derkarabetian, S. , L. R. Benavides , and G. Giribet . 2019. “Sequence Capture Phylogenomics of Historical Ethanol‐Preserved Museum Specimens: Unlocking the Rest of the Vault.” Molecular Ecology Resources 19, no. 6: 1531–1544. 31448547 10.1111/1755-0998.13072

[eva70195-bib-0028] Dokan, K. , S. Kawamura , and K. M. Teshima . 2021. “Effects of Single Nucleotide Polymorphism Ascertainment on Population Structure Inferences.” G3: Genes, Genomes, Genetics 11, no. 9: jkab128. 33871576 10.1093/g3journal/jkab128 PMC8496283

[eva70195-bib-0029] Erickson, K. L. , A. Pentico , A. M. Quattrini , and C. S. McFadden . 2021. “New Approaches to Species Delimitation and Population Structure of Anthozoans: Two Case Studies of Octocorals Using Ultraconserved Elements and Exons.” Molecular Ecology Resources 21, no. 1: 78–92. 32786110 10.1111/1755-0998.13241

[eva70195-bib-0140] Everett, M. V. , L. K. Park , E. A. Berntson , et al. 2016. “Large‐Scale Genotyping‐by‐Sequencing Indicates High Levels of Gene Flow in the Deep‐Sea Octocoral *Swiftia simplex* (Nutting 1909) on the West Coast of the United States.” PLoS One 11, no. 10: e0165279. 10.1371/journal.pone.0165279. 27798660 PMC5087884

[eva70195-bib-0030] Faircloth, B. C. 2013. “Illumiprocessor: A Trimmomatic Wrapper for Parallel Adapter and Quality Trimming.” *github*. 10.6079/J9ILL.

[eva70195-bib-0031] Faircloth, B. C. 2016. “PHYLUCE Is a Software Package for the Analysis of Conserved Genomic Loci.” Bioinformatics 32, no. 5: 786–788. 26530724 10.1093/bioinformatics/btv646

[eva70195-bib-0137] Faircloth, B. C. , J. E. McCormack , N. G. Crawford , M. G. Harvey , R. T. Brumfield , and T. C. Glenn . 2012. “Ultraconserved Elements Anchor Thousands of Genetic Markers Spanning Multiple Evolutionary Timescales.” Systematic Biology 61, no. 5: 717–726. 10.1093/sysbio/sys004. 22232343

[eva70195-bib-0032] Feng, D. , and D. Chen . 2015. “Authigenic Carbonates From an Active Cold Seep of the Northern South China Sea: New Insights Into Fluid Sources and Past Seepage Activity.” Deep Sea Research. Part II, Topical Studies in Oceanography 122: 74–83.

[eva70195-bib-0033] Fitak, R. R. 2021. “OptM: Estimating the Optimal Number of Migration Edges on Population Trees Using Treemix.” Biology Methods & Protocols 6, no. 1: bpab017. 34595352 10.1093/biomethods/bpab017 PMC8476930

[eva70195-bib-0034] Foll, M. , and O. Gaggiotti . 2008. “A Genome‐Scan Method to Identify Selected Loci Appropriate for Both Dominant and Codominant Markers: A Bayesian Perspective.” Genetics 180, no. 2: 977–993. 18780740 10.1534/genetics.108.092221 PMC2567396

[eva70195-bib-0035] Francis, R. M. 2017. “Pophelper: An R Package and Web App to Analyse and Visualize Population Structure.” Molecular Ecology Resources 17, no. 1: 27–32. 26850166 10.1111/1755-0998.12509

[eva70195-bib-0036] Fu, L. , C. Cai , Y. Cui , et al. 2016. “Pooled Mapping: An Efficient Method of Calling Variations for Population Samples With Low‐Depth Resequencing Data.” Molecular Breeding 36, no. 4: 1–12.

[eva70195-bib-0037] Fu, L. , B. Niu , Z. Zhu , S. Wu , and W. Li . 2012. “CD‐HIT: Accelerated for Clustering the Next‐Generation Sequencing Data.” Bioinformatics 28, no. 23: 3150–3152. 23060610 10.1093/bioinformatics/bts565 PMC3516142

[eva70195-bib-0038] Gaither, M. R. , G. A. Gkafas , M. de Jong , et al. 2018. “Genomics of Habitat Choice and Adaptive Evolution in a Deep‐Sea Fish.” Nature Ecology & Evolution 2, no. 4: 680–687. 29507380 10.1038/s41559-018-0482-x

[eva70195-bib-0039] Glon, H. , A. Quattrini , E. Rodríguez , B. M. Titus , and M. Daly . 2021. “Comparison of Sequence‐Capture and ddRAD Approaches in Resolving Species and Populations in Hexacorallian Anthozoans.” Molecular Phylogenetics and Evolution 163: 107233. 34139346 10.1016/j.ympev.2021.107233

[eva70195-bib-0040] Gordon, J. 2023. Population and Seascape Genomics of the Deep‐Sea Octocoral *Acanella arbuscula* .” Doctoral Thesis. University of Essex.

[eva70195-bib-0041] Goudet, J. 2005. “Hierfstat, a Package for r to Compute and Test Hierarchical F‐Statistics.” Molecular Ecology Notes 5, no. 1: 184–186.

[eva70195-bib-0042] Grosell, M. , E. M. Mager , C. Williams , and J. R. Taylor . 2009. “High Rates of HCO_3_ ^−^ Secretion and cl^−^ Absorption Against Adverse Gradients in the Marine Teleost Intestine: The Involvement of an Electrogenic Anion Exchanger and H^+^‐Pump Metabolon?” Journal of Experimental Biology 212, no. 11: 1684–1696. 19448078 10.1242/jeb.027730

[eva70195-bib-0043] Gutenkunst, R. N. , R. D. Hernandez , S. H. Williamson , and C. D. Bustamante . 2009. “Inferring the Joint Demographic History of Multiple Populations From Multidimensional SNP Data.” PLoS Genetics 5, no. 10: e1000695. 19851460 10.1371/journal.pgen.1000695 PMC2760211

[eva70195-bib-0045] Harvey, M. G. , B. T. Smith , T. C. Glenn , B. C. Faircloth , and R. T. Brumfield . 2016. “Sequence Capture Versus Restriction Site Associated DNA Sequencing for Shallow Systematics.” Systematic Biology 65, no. 5: 910–924. 27288477 10.1093/sysbio/syw036

[eva70195-bib-0046] He, J. , Z. Wu , L. Chen , et al. 2021. “Adenosine Triggers Larval Settlement and Metamorphosis in the Mussel *Mytilopsis sallei* Through the ADK‐AMPK‐FoxO Pathway.” ACS Chemical Biology 16, no. 8: 1390–1400. 34254778 10.1021/acschembio.1c00175

[eva70195-bib-0047] He, X. , T. Xu , C. Chen , et al. 2023. “Same (Sea) Bed Different Dreams: Biological Community Structure of the Haima Seep Reveals Distinct Biogeographic Affinities.” Innovation Geoscience 1, no. 2: 100019.

[eva70195-bib-0048] Hoegg, S. , and A. Meyer . 2007. “Phylogenomic Analyses of KCNA Gene Clusters in Vertebrates: Why Do Gene Clusters Stay Intact?” BMC Evolutionary Biology 7, no. 1: 139. 17697377 10.1186/1471-2148-7-139 PMC1978502

[eva70195-bib-0049] Hohenlohe, P. A. , S. J. Amish , J. M. Catchen , F. W. Allendorf , and G. Luikart . 2011. “Next‐Generation RAD Sequencing Identifies Thousands of SNPs for Assessing Hybridization Between Rainbow and Westslope Cutthroat Trout.” Molecular Ecology Resources 11, no. Suppl 1: 117–122. 21429168 10.1111/j.1755-0998.2010.02967.x

[eva70195-bib-0050] Hohenlohe, P. A. , W. C. Funk , and O. P. Rajora . 2021. “Population Genomics for Wildlife Conservation and Management.” Molecular Ecology 30, no. 1: 62–82. 33145846 10.1111/mec.15720 PMC7894518

[eva70195-bib-0052] Jombart, T. 2008. “Adegenet: A R Package for the Multivariate Analysis of Genetic Markers.” Bioinformatics 24, no. 11: 1403–1405. 18397895 10.1093/bioinformatics/btn129

[eva70195-bib-0053] Kern, A. D. , D. A. Barbash , J. Chang Mell , D. Hupalo , and A. Jensen . 2015. “Highly Constrained Intergenic *Drosophila* Ultraconserved Elements Are Candidate ncRNAs.” Genome Biology and Evolution 7, no. 3: 689–698. 25618141 10.1093/gbe/evv011 PMC5322558

[eva70195-bib-0054] Knaus, B. J. , and N. J. Grünwald . 2017. “Vcfr: A Package to Manipulate and Visualize Variant Call Format Data in R.” Molecular Ecology Resources 17, no. 1: 44–53. 27401132 10.1111/1755-0998.12549

[eva70195-bib-0055] Korunes, K. L. , and K. Samuk . 2021. “Pixy: Unbiased Estimation of Nucleotide Diversity and Divergence in the Presence of Missing Data.” Molecular Ecology Resources 21, no. 4: 1359–1368. 33453139 10.1111/1755-0998.13326 PMC8044049

[eva70195-bib-0056] Kyuno, A. , M. Shintaku , Y. Fujita , et al. 2009. “Dispersal and Differentiation of Deep‐Sea Mussels of the Genus *Bathymodiolus* (Mytilidae, Bathymodiolinae).” Journal of Marine Sciences 2009, no. 1: e625672.

[eva70195-bib-0057] Lemmon, A. R. , S. A. Emme , and E. M. Lemmon . 2012. “Anchored Hybrid Enrichment for Massively High‐Throughput Phylogenomics.” Systematic Biology 61, no. 5: 727–744. 22605266 10.1093/sysbio/sys049

[eva70195-bib-0059] Li, H. 2013. “Aligning Sequence Reads, Clone Sequences and Assembly Contigs With BWA‐MEM.” *arXiv*, 1303.3997.

[eva70195-bib-0060] Li, L. , A. Li , K. Song , et al. 2018. “Divergence and Plasticity Shape Adaptive Potential of the Pacific Oyster.” Nature Ecology & Evolution 2, no. 11: 1751–1760. 30250157 10.1038/s41559-018-0668-2

[eva70195-bib-0061] Li, Y. X. 2025. “Supplementary Files: Population Genomics of Deep‐Sea Mussels Using UCEs.” *FigShare*. 10.6084/m9.figshare.26215427.

[eva70195-bib-0062] Li, Y. X. , J.C.‐H. Ip , C. Chen , et al. 2025. “Phylogenomics of Bivalvia Using Ultraconserved Elements (UCEs) Reveal New Topologies for Pteriomorphia and Imparidentia.” Systematic Biology 74, no. 1: 16–33. 39283716 10.1093/sysbio/syae052

[eva70195-bib-0063] Li, Y. X. , Y. Sun , Y. T. Lin , T. Xu , J. C. H. Ip , and J. W. Qiu . 2023. “Cold Seep Macrofauna.” In South China Sea Seeps, 69–88. Springer.

[eva70195-bib-0064] Lim, H. C. , and M. J. Braun . 2016. “High‐Throughput SNP Genotyping of Historical and Modern Samples of Five Bird Species via Sequence Capture of Ultraconserved Elements.” Molecular Ecology Resources 16, no. 5: 1204–1223. 27427784 10.1111/1755-0998.12568

[eva70195-bib-0065] Lim, H. C. , S. B. Shakya , M. G. Harvey , et al. 2020. “Opening the Door to Greater Phylogeographic Inference in Southeast Asia: Comparative Genomic Study of Five Codistributed Rainforest Bird Species Using Target Capture and Historical DNA.” Ecology and Evolution 10, no. 7: 3222–3247. 32273983 10.1002/ece3.5964 PMC7141000

[eva70195-bib-0066] Lischer, H. E. L. , and L. Excoffier . 2012. “PGDSpider: An Automated Data Conversion Tool for Connecting Population Genetics and Genomics Programs.” Bioinformatics 28, no. 2: 298–299. 22110245 10.1093/bioinformatics/btr642

[eva70195-bib-0067] Liu, X. , and Y.‐X. Fu . 2020. “Stairway Plot 2: Demographic History Inference With Folded SNP Frequency Spectra.” Genome Biology 21, no. 1: 280. 33203475 10.1186/s13059-020-02196-9 PMC7670622

[eva70195-bib-0068] Liu, Z. , Y. Huang , H. Chen , et al. 2023. “Chromosome‐Level Genome Assembly of the Deep‐Sea Snail *Phymorhynchus buccinoides* Provides Insights Into the Adaptation to the Cold Seep Habitat.” BMC Genomics 24, no. 1: 679. 37950158 10.1186/s12864-023-09760-0 PMC10638732

[eva70195-bib-0069] Lu, C. , X. Qin , J. Sun , R. Wang , and J. Cai . 2023. “Research Progress and Scientific Challenges in the Depressurization Exploitation Mechanism of Clayey‐Silt Natural Gas Hydrates in the Northern South China Sea.” Advances in Geo‐Energy Research 10, no. 1: 14–20.

[eva70195-bib-0070] Luo, J. , T. Liao , C. Yang , et al. 2024. “RNA‐Seq and Metabolomic Analysis Reveal Dynamic Response Mechanism to Hypoxia in the Pearl Oyster *Pinctada fucata martensii* .” Regional Studies in Marine Science 77: 103725.

[eva70195-bib-0071] Ma, H. , W. Dellisanti , J. T. H. Chung , et al. 2025. “Proteomic Insights Into the Environmental Adaptation of the Subtropical Brain Coral Host *Platygyra carnosa* .” iScience 28, no. 4: 112287. 40248114 10.1016/j.isci.2025.112287 PMC12005889

[eva70195-bib-0072] Mao, J. , Y. Tian , Q. Liu , et al. 2024. “Revealing Genetic Diversity, Population Structure, and Selection Signatures of the Pacific Oyster in Dalian by Whole‐Genome Resequencing.” Frontiers in Ecology and Evolution 11: 1337980.

[eva70195-bib-0073] Mapel, X. M. , E. F. Gyllenhaal , T. H. Modak , et al. 2021. “Inter‐ and Intra‐Archipelago Dynamics of Population Structure and Gene Flow in a Polynesian Bird.” Molecular Phylogenetics and Evolution 156: 107034. 33276120 10.1016/j.ympev.2020.107034

[eva70195-bib-0074] McCole, R. B. , J. Erceg , W. Saylor , and C. Wu . 2018. “Ultraconserved Elements Occupy Specific Arenas of Three‐Dimensional Mammalian Genome Organization.” Cell Reports 24, no. 2: 479–488. 29996107 10.1016/j.celrep.2018.06.031 PMC6363003

[eva70195-bib-0138] McCormack, J. E. , B. C. Faircloth , N. G. Crawford , P. A. Gowaty , R. T. Brumfield , and T. C. Glenn . 2011. “Ultraconserved Elements Are Novel Phylogenomic Markers That Resolve Placental Mammal Phylogeny When Combined with Species‐Tree Analysis.” Genome Research 22, no. 4: 746–754. 10.1101/gr.125864.111. 22207614 PMC3317156

[eva70195-bib-0075] McKenna, A. , M. Hanna , E. Banks , et al. 2010. “The Genome Analysis Toolkit: A MapReduce Framework for Analyzing Next‐Generation DNA Sequencing Data.” Genome Research 20, no. 9: 1297–1303. 20644199 10.1101/gr.107524.110 PMC2928508

[eva70195-bib-0076] Mikheenko, A. , A. Prjibelski , V. Saveliev , D. Antipov , and A. Gurevich . 2018. “Versatile Genome Assembly Evaluation With QUAST‐LG.” Bioinformatics 34, no. 13: i142–i150. 29949969 10.1093/bioinformatics/bty266 PMC6022658

[eva70195-bib-0077] Miyazaki, J.‐I. , S. Beppu , S. Kajio , et al. 2013. “Dispersal Ability and Environmental Adaptability of Deep‐Sea Mussels *Bathymodiolus* (Mytilidae: Bathymodiolinae).” Open Journal of Marine Science 3: 27168.

[eva70195-bib-0078] Myers, E. A. , R. W. Bryson Jr. , R. W. Hansen , M. L. Aardema , D. Lazcano , and F. T. Burbrink . 2019. “Exploring Chihuahuan Desert Diversification in the Gray‐Banded Kingsnake, *Lampropeltis alterna* (Serpentes: Colubridae).” Molecular Phylogenetics and Evolution 131: 211–218. 30389598 10.1016/j.ympev.2018.10.031

[eva70195-bib-0080] Nurk, S. , A. Bankevich , D. Antipov , et al. 2013. “Assembling Genomes and Mini‐Metagenomes From Highly Chimeric Reads.” Lecture Notes in Computer Science 7821: 158–170.

[eva70195-bib-0081] Okamato, N. , S. Shiokawa , S. Kawano , N. Yamaji , H. Sakurai , and M. Kurihara . 2019. “World's First Lifting Test for Seafloor Massive Sulphides in Okinawa Trough in the EEZ of Japan.” *The 29th International Ocean and Polar Engineering Conference*.

[eva70195-bib-0082] Oleksiak, M. F. , and O. P. Rajora . 2020. “Marine Population Genomics: Challenges and Opportunities.” In Population Genomics: Marine Organisms, edited by M. F. Oleksiak and O. P. Rajora , 3–35. Springer International Publishing.

[eva70195-bib-0083] Ortiz‐Sepulveda, C. M. , M. Genete , C. Blassiau , et al. 2023. “Target Enrichment of Long Open Reading Frames and Ultraconserved Elements to Link Microevolution and Macroevolution in Non‐Model Organisms.” Molecular Ecology Resources 23, no. 3: 659–679. 36349833 10.1111/1755-0998.13735

[eva70195-bib-0085] Ou, J. , and L. J. Zhu . 2019. “trackViewer: A Bioconductor Package for Interactive and Integrative Visualization of Multi‐Omics Data.” Nature Methods 16, no. 6: 453–454. 31133757 10.1038/s41592-019-0430-y

[eva70195-bib-0087] Petersen, H. C. , B. W. Hansen , K. E. Knott , and G. T. Banta . 2022. “Species and Genetic Diversity Relationships in Benthic Macroinvertebrate Communities Along a Salinity Gradient.” BMC Ecology and Evolution 22, no. 1: 125. 36324063 10.1186/s12862-022-02087-6 PMC9632067

[eva70195-bib-0088] Petersen, H. C. , K. E. Knott , G. T. Banta , and B. W. Hansen . 2022. “Ultra‐Conserved Elements Provide Insights to the Biogeographic Patterns of Three Benthic Macroinvertebrate Species in the Baltic Sea.” Estuarine, Coastal and Shelf Science 271: 107863.

[eva70195-bib-0089] Pickrell, J. K. , and J. K. Pritchard . 2012. “Inference of Population Splits and Mixtures From Genome‐Wide Allele Frequency Data.” PLoS Genetics 8, no. 11: e1002967. 23166502 10.1371/journal.pgen.1002967 PMC3499260

[eva70195-bib-0141] Ponder, W. F. , D. R. Lindberg , and J. M. Ponder . 2020. Biology and Evolution of the Mollusca. CRC Press. 10.1201/9781351115254.

[eva70195-bib-0090] Portanier, E. , A. Nicolle , W. Rath , et al. 2023. “Coupling Large‐Spatial Scale Larval Dispersal Modelling With Barcoding to Refine the Amphi‐Atlantic Conectivity Hypothesis in Deep‐Sea Seep Mussels.” Frontiers in Marine Science 10: 1122124.

[eva70195-bib-0091] Purcell, S. , B. Neale , K. Todd‐Brown , et al. 2007. “PLINK: A Tool Set for Whole‐Genome Association and Population‐Based Linkage Analyses.” American Journal of Human Genetics 81, no. 3: 559–575. 17701901 10.1086/519795 PMC1950838

[eva70195-bib-0092] Puritz, J. B. , C. M. Hollenbeck , and J. R. Gold . 2014. “dDocent: A RADseq, Variant‐Calling Pipeline Designed for Population Genomics of Non‐Model Organisms.” PeerJ 2014, no. 1: e431. 10.7717/peerj.431 PMC4060032 24949246

[eva70195-bib-0093] Qi, H. , L. Li , and G. Zhang . 2021. “Construction of a Chromosome‐Level Genome and Variation Map for the Pacific Oyster *Crassostrea gigas* .” Molecular Ecology Resources 21, no. 5: 1670–1685. 33655634 10.1111/1755-0998.13368

[eva70195-bib-0094] Ritchie, H. , A. J. Jamieson , and S. B. Piertney . 2016. “Isolation and Characterization of Microsatellite DNA Markers in the Deep‐Sea Amphipod *Paralicella tenuipes* by Illumina MiSeq Sequencing.” Journal of Heredity 107, no. 4: 367–371. 27012615 10.1093/jhered/esw019 PMC4888441

[eva70195-bib-0095] Shen, Y. , Q. Kou , W. Chen , et al. 2016. “Comparative Population Structure of Two Dominant Species, *Shinkaia crosnieri* (Munidopsidae: *Shinkaia*) and *Bathymodiolus platifrons* (Mytilidae: Bathymodiolus), Inhabiting Both Deep‐Sea Vent and Cold Seep Inferred From Mitochondrial Multi‐Genes.” Ecology and Evolution 6, no. 11: 3571–3582. 28725351 10.1002/ece3.2132 PMC5513293

[eva70195-bib-0096] Sigwart, J. D. , D. R. Lindberg , C. Chen , and J. Sun . 2021. “Molluscan Phylogenomics Requires Strategically Selected Genomes.” Philosophical Transactions of the Royal Society B 376, no. 1825: 20200161. 10.1098/rstb.2020.0161 PMC8059523 33813889

[eva70195-bib-0097] Singhal, S. , M. R. Grundler , G. R. Colli , and D. L. Rabosky . 2017. “Squamate Conserved Loci (SqCL): A Unified Set of Conserved Loci for Phylogenomics and Population Genetics of Squamate Reptiles.” Molecular Ecology Resources 17, no. 6: 593–614. 10.1111/1755-0998.12681 28417603

[eva70195-bib-0099] Snetkova, V. , L. A. Pennacchio , A. Visel , and D. E. Dickel . 2022. “Perfect and Imperfect Views of Ultraconserved Sequences.” Nature Reviews Genetics 23, no. 3: 182–194. 10.1038/s41576-021-00424-x PMC8858888 34764456

[eva70195-bib-0100] Stange, M. , R. D. H. Barrett , and A. P. Hendry . 2020. “The Importance of Genomic Variation for Biodiversity, Ecosystems and People.” Nature Reviews Genetics 22, no. 2: 89–105. 10.1038/s41576-020-00288-7 33067582

[eva70195-bib-0101] Stiller, J. , R. R. da Fonseca , M. E. Alfaro , B. C. Faircloth , N. G. Wilson , and G. W. Rouse . 2021. “Using Ultraconserved Elements to Track the Influence of Sea‐Level Change on Leafy Sea Dragon Populations.” Molecular Ecology 30, no. 6: 1364–1380. 33217068 10.1111/mec.15744

[eva70195-bib-0102] Sun, J. , Y. Zhang , T. Xu , et al. 2017. “Adaptation to Deep‐Sea Chemosynthetic Environments as Revealed by Mussel Genomes.” Nature Ecology & Evolution 1, no. 5: 0121. 10.1038/s41559-017-0121 28812709

[eva70195-bib-0103] Taylor, M. L. , and C. N. Roterman . 2017. “Invertebrate Population Genetics Across Earth's Largest Habitat: The Deep‐Sea Floor.” Molecular Ecology 26, no. 19: 4872–4896. 28833857 10.1111/mec.14237

[eva70195-bib-0104] Thacker, C. E. , J. J. Shelley , W. T. McCraney , M. Adams , M. P. Hammer , and P. J. Unmack . 2022. “Phylogeny, Diversification, and Biogeography of a Hemiclonal Hybrid System of Native Australian Freshwater Fishes (Gobiiformes: Gobioidei: Eleotridae: *Hypseleotris*).” BMC Ecology and Evolution 22, no. 1: 22. 35236294 10.1186/s12862-022-01981-3 PMC8892812

[eva70195-bib-0105] Theissinger, K. , C. Fernandes , G. Formenti , et al. 2023. “How Genomics Can Help Biodiversity Conservation.” Trends in Genetics 39, no. 7: 545–559. 36801111 10.1016/j.tig.2023.01.005

[eva70195-bib-0106] Thorburn, D. M. J. , K. Sagonas , M. Binzer‐Panchal , et al. 2023. “Origin Matters: Using a Local Reference Genome Improves Measures in Population Genomics.” Molecular Ecology Resources 23, no. 7: 1706–1723. 37489282 10.1111/1755-0998.13838

[eva70195-bib-0108] Tran, L. Y. A. , S. Ruault , C. Daguin‐Thiébaut , et al. 2022. “Subtle Limits to Connectivity Revealed by Outlier Loci Within Two Divergent Metapopulations of the Deep‐Sea Hydrothermal Gastropod *Ifremeria nautilei* .” Molecular Ecology 31, no. 10: 2796–2813. 35305041 10.1111/mec.16430

[eva70195-bib-0109] Van Dam, M. H. , J. B. Henderson , L. Esposito , and M. Trautwein . 2021. “Genomic Characterization and Curation of UCEs Improves Species Tree Reconstruction.” Systematic Biology 70, no. 2: 307–321. 32750133 10.1093/sysbio/syaa063 PMC7875437

[eva70195-bib-0110] van der Reis, A. L. , C. R. Norrie , A. G. Jeffs , S. D. Lavery , and E. L. Carroll . 2022. “Genetic and Particle Modelling Approaches to Assessing Population Connectivity in a Deep Sea Lobster.” Scientific Reports 12, no. 1: 16783. 36202873 10.1038/s41598-022-19790-5 PMC9537507

[eva70195-bib-0111] Vendrami, D. L. J. , L. Telesca , H. Weigand , et al. 2017. “RAD Sequencing Resolves Fine‐Scale Population Structure in a Benthic Invertebrate: Implications for Understanding Phenotypic Plasticity.” Royal Society Open Science 4, no. 2: 160548. 28386419 10.1098/rsos.160548 PMC5367306

[eva70195-bib-0112] Vinciguerra, N. T. , W. L. E. Tsai , B. C. Faircloth , and J. E. McCormack . 2019. “Comparison of Ultraconserved Elements (UCEs) to Microsatellite Markers for the Study of Avian Hybrid Zones: A Test in *Aphelocoma* Jays.” BMC Research Notes 12, no. 1: 456. 31340859 10.1186/s13104-019-4481-z PMC6657088

[eva70195-bib-0113] Virtanen, P. , R. Gommers , T. E. Oliphant , et al. 2020. “SciPy 1.0: Fundamental Algorithms for Scientific Computing in Python.” Nature Methods 17, no. 3: 261–272. 32015543 10.1038/s41592-019-0686-2 PMC7056644

[eva70195-bib-0114] Wang, H. , L. Pan , L. Si , R. Ji , and Y. Cao . 2021. “Effects of Nrf2‐Keap1 Signaling Pathway on Antioxidant Defense System and Oxidative Damage in the Clams *Ruditapes philippinarum* Exposure to PAHs.” Environmental Science and Pollution Research 28, no. 25: 33060–33071. 10.1007/s11356-021-12906-w 33638075

[eva70195-bib-0115] Washburn, T. W. , A. Iguchi , K. Yamaoka , et al. 2023. “Impacts of the First Deep‐Sea Seafloor Massive Sulfide Mining Excavation Tests on Benthic Communities.” Marine Ecology Progress Series 712: 1–19.

[eva70195-bib-0116] Watanabe, H. , K. Fujikura , S. Kojima , J.‐I. Miyazaki , and Y. Fujiwara . 2010. “Japan: Vents and Seeps in Close Proximity.” In Topics in Geobiology. Springer Japan.

[eva70195-bib-0117] Wilkinson, L. 2011. “ggplot2: Elegant Graphics for Data Analysis by WICKHAM, H.” Biometrics 67, no. 2: 678–679.

[eva70195-bib-0118] Winker, K. , T. C. Glenn , and B. C. Faircloth . 2018. “Ultraconserved Elements (UCEs) Illuminate the Population Genomics of a Recent, High‐Latitude Avian Speciation Event.” PeerJ 6: e5735. 30310754 10.7717/peerj.5735 PMC6174879

[eva70195-bib-0119] Xiao, Y. , T. Xu , J. Sun , et al. 2020. “Population Genetic Structure and Gene Expression Pplasticity of the Deep‐Sea Vent and Seep Squat Lobster *Shinkaia crosnieri* .” Frontiers in Marine Science 7: 587686.

[eva70195-bib-0120] Xu, T. , X. Chai , C. Chen , et al. 2024. “Genetic Divergence and Migration Patterns of a Galatheoid Squat Lobster Highlight the Need for Deep‐Sea Conservation.” Molecular Ecology 33, no. 1: e17200. 37985390 10.1111/mec.17200

[eva70195-bib-0121] Xu, T. , J. Sun , J. Lv , et al. 2017. “Genome‐Wide Discovery of Single Nucleotide Polymorphisms (SNPs) and Single Nucleotide Variants (SNVs) in Deep‐Sea Mussels: Potential Use in Population Genomics and Cross‐Species Application.” Deep Sea Research. Part II, Topical Studies in Oceanography 137: 318–326.

[eva70195-bib-0122] Xu, T. , J. Sun , H. K. Watanabe , et al. 2018. “Population Genetic Structure of the Deep‐Sea Mussel *Bathymodiolus platifrons* (Bivalvia: Mytilidae) in the Northwest Pacific.” Evolutionary Applications 11, no. 10: 1915–1930. 30459838 10.1111/eva.12696 PMC6231483

[eva70195-bib-0123] Xu, T. , Y. Wang , J. Sun , et al. 2021. “Hidden Historical Habitat‐Linked Population Divergence and Contemporary Gene Flow of a Deep‐Sea Patellogastropod Limpet.” Molecular Biology and Evolution 38, no. 12: 5640–5654. 34534352 10.1093/molbev/msab278 PMC8662656

[eva70195-bib-0125] Yue, C. , Q. Li , H. Yu , S. Liu , and L. Kong . 2020. “Restriction Site‐Associated DNA Sequencing (RAD‐Seq) Analysis in Pacific Oyster *Crassostrea gigas* Based on Observation of Individual Sex Changes.” Scientific Reports 10, no. 1: 9873. 32555506 10.1038/s41598-020-67007-4 PMC7303127

[eva70195-bib-0126] Zarza, E. , B. C. Faircloth , W. L. E. Tsai , R. W. Bryson Jr. , J. Klicka , and J. E. McCormack . 2016. “Hidden Histories of Gene Flow in Highland Birds Revealed With Genomic Markers.” Molecular Ecology 25, no. 20: 5144–5157. 27543758 10.1111/mec.13813

[eva70195-bib-0127] Zeng, L. , Y.‐H. Wang , C.‐X. Ai , et al. 2024. “Differential Effects of Oxytetracycline on Detoxification and Antioxidant Defense in the Hepatopancreas and Intestine of Chinese Mitten Crab Under Cadmium Stress.” Science of the Total Environment 930: 172633. 38643877 10.1016/j.scitotenv.2024.172633

[eva70195-bib-0128] Zhang, L. , W. Sun , H. Chen , F. Tian , and W. Cai . 2020. “Transcriptome Analysis of Acute Exposure of the Manila Clam, *Ruditapes philippinarum* to Pperfluorooctane Sulfonate (PFOS).” Comparative Biochemistry and Physiology Part C, Toxicology & Pharmacology 231: 108736. 32142923 10.1016/j.cbpc.2020.108736

[eva70195-bib-0129] Zhang, P. , X. Zeng , J. Fu , and Y. Zheng . 2021. “UCE Phylogenomics, Detection of a Putative Hybrid Population, and One Older Mitogenomic Node Age of *Batrachuperus salamanders* .” Molecular Phylogenetics and Evolution 163: 107239. 34214665 10.1016/j.ympev.2021.107239

[eva70195-bib-0130] Zhang, W. , J. Liang , Q. Liang , et al. 2021. “Gas Hydrate Accumulation and Occurrence Associated With Cold Seep Systems in the Northern South China Sea: An Overview.” Geofluids 2021, no. 1: 5571150.

[eva70195-bib-0131] Zhang, X. , Y. Tian , H. Yu , M. Cao , and C. Li . 2023. “Genome‐Wide Characterization of Mapk Gene Family in Black Rockfish *Sebastes schlegelii* and Their Expression Patterns Against *Edwardsiella piscicida* Infection.” Journal of Oceanology and Limnology 41, no. 6: 2348–2362.

[eva70195-bib-0132] Zhang, Y. , J. Cheng , Z. Sha , and M. Hui . 2024. “Population Genetic Structure and Implication for Adaptive Differentiation of the Snail (Gastropoda, Provannidae) in Deep‐Sea Chemosynthetic Ecosystems.” Zoologica Scripta 53, no. 2: 192–206.

[eva70195-bib-0133] Zhang, Z. , M. A. Miteva , L. Wang , and E. Alexov . 2012. “Analyzing Effects of Naturally Occurring Missense Mutations.” Computational and Mathematical Methods in Medicine 2012, no. 1: 805827. 22577471 10.1155/2012/805827 PMC3346971

[eva70195-bib-0134] Zhao, X. , H. Yu , L. Kong , and Q. Li . 2012. “Transcriptomic Responses to Salinity Stress in the Pacific Oyster *Crassostrea gigas* .” PLoS One 7, no. 9: e46244. 23029449 10.1371/journal.pone.0046244 PMC3459877

[eva70195-bib-0135] Zhao, Y. , T. Xu , Y. S. Law , et al. 2020. “Ecological Characterization of Cold‐Seep Epifauna in the South China Sea.” Deep‐Sea Research Part I: Oceanographic Research Papers 163: 103361.

[eva70195-bib-0136] Zheng, M. , T. Cierpicki , A. J. Burdette , et al. 2011. “Structural Features and Chaperone Activity of the NudC Protein Family.” Journal of Molecular Biology 409, no. 5: 722–741. 21530541 10.1016/j.jmb.2011.04.018 PMC3159028

