# Supplementary material for: Sequencing Ultraconserved Elements (UCEs) for Marine Population Genomics: A Proof‐of‐Concept Using a Deep‐Sea Mussel Species
Source: Evol Appl. 2026 Jan 13;19(1):e70195. doi: 10.1111/eva.70195 (PMC12797253; doi:10.1111/eva.70195)

**Supplementary figures**

**Table of Contents:**

| **Figure S1** | Page 2 |
| --- | --- |
| **Figure S2** | Page 3 |
| **Figure S3** | Page 4 |
| **Figure S4** | Page 5 |
| **Figure S5** | Page 6 |
| **Figure S6** | Page 7 |
| **Figure S7** | Page 8 |
| **Figure S8** | Page 9 |
| **Figure S9** | Page 10 |
| **Figure S10** | Page 11 |
| **Figure S11** | Page 12 |
| **Figure S12** | Page 13 |
| **Figure S13** | Page 14 |
| **Figure S14** | Page 15 |
| **Figure S15** | Page 16 |
| **Figure S16** | Page 17 |
| **Figure S17** | Page 18 |
| **Figure S18** | Page 19 |
| **Figure S19** | Page 20 |
| **Figure S20** | Page 21 |
| **Figure S21** | Page 22 |
| **Figure S22** | Page 23 |
| **Figure S23** | Page 24 |

**Fig. S1** Summary of site depth for JR population based on vcf1 (1× – 30 ×).

**
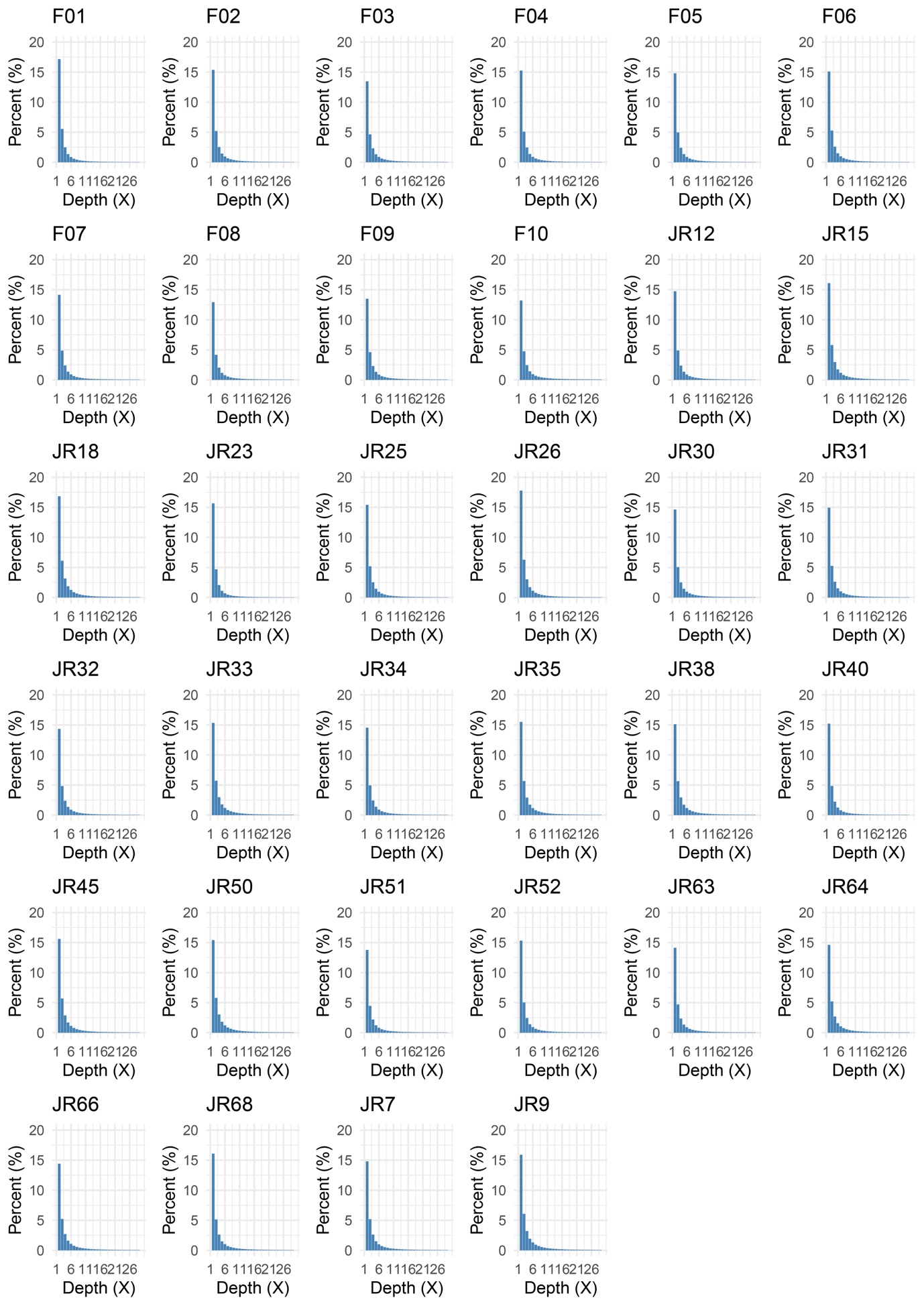
**

**Fig. S2** Summary of site depth for SB population based on vcf1 (1× – 30 ×).

**
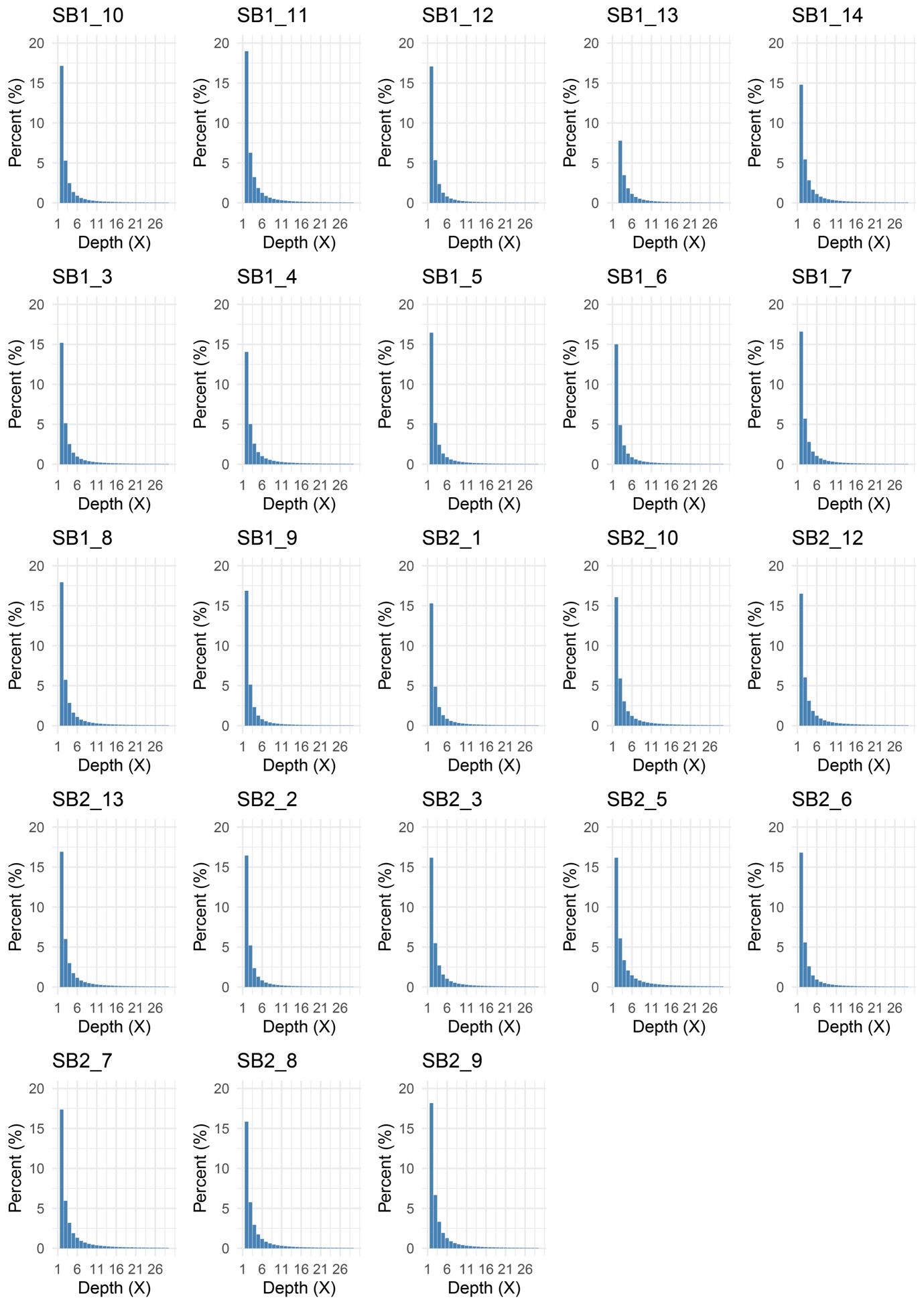
**

**Fig. S3** Summary of site depth for DK population based on vcf1 (1× – 30 ×).

**
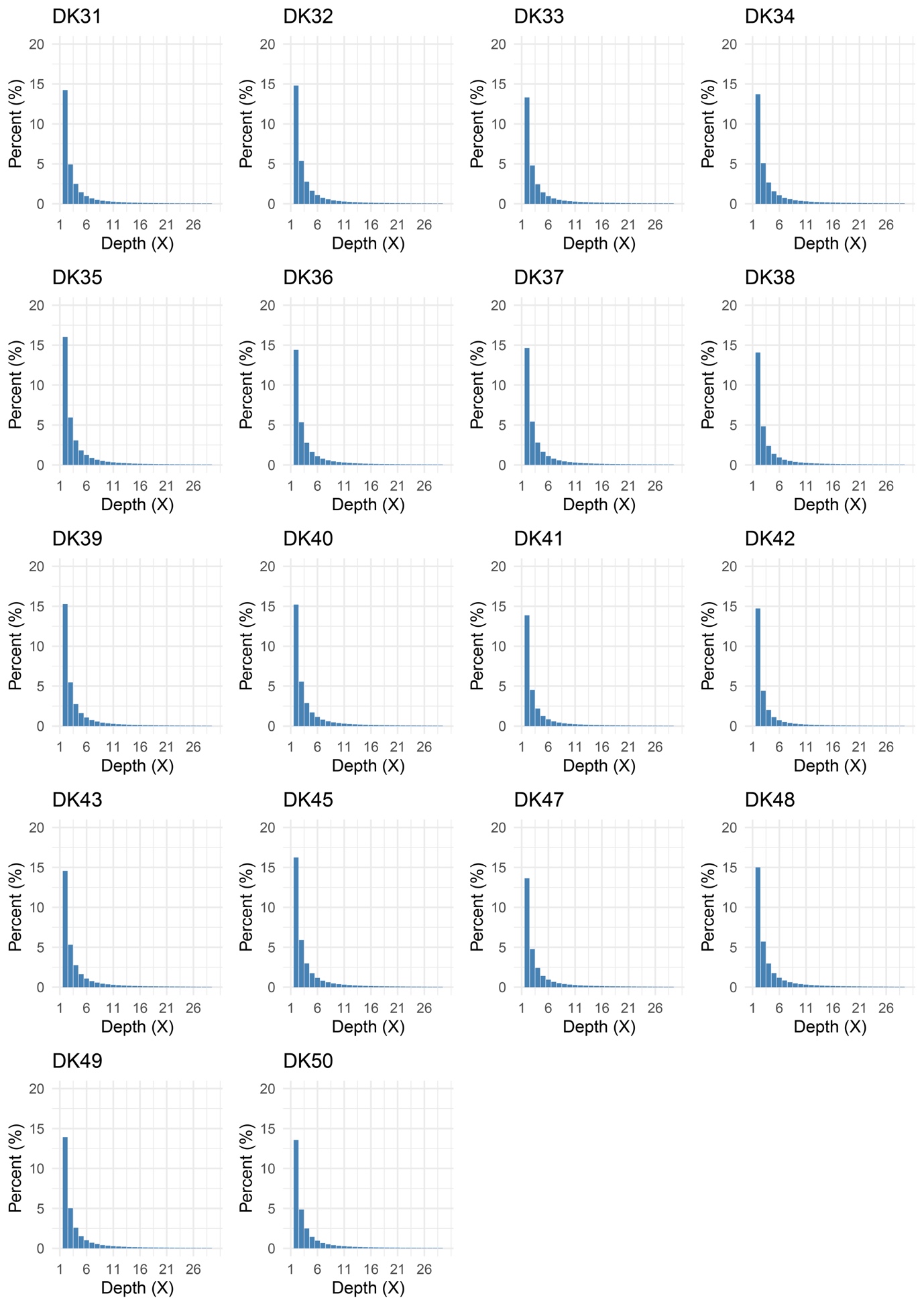
**

**Fig. S4** Summary of site depth for HK population based on vcf1 (1× – 30 ×).

**
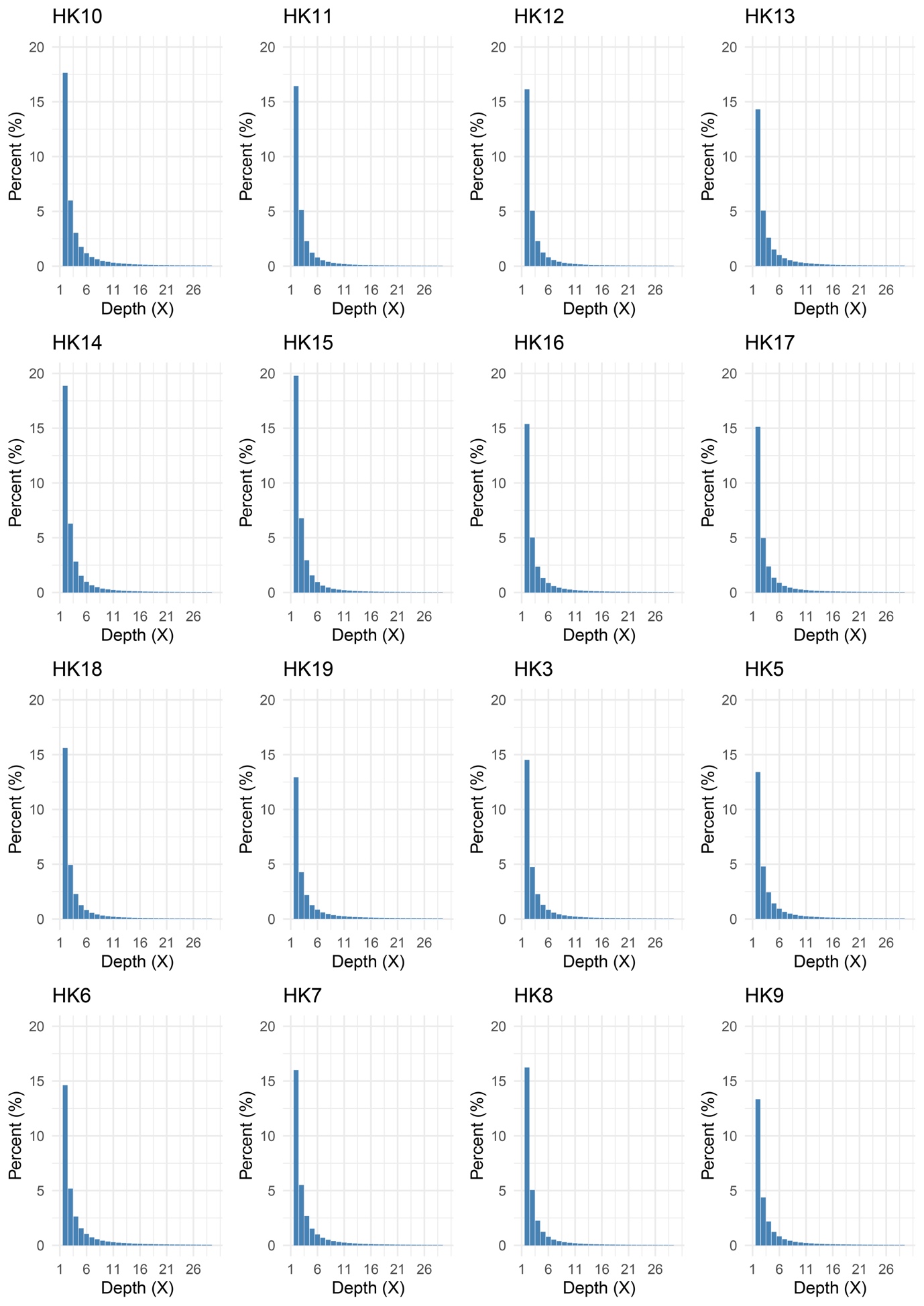
**

**Fig. S5** Summary of site depth for IR population based on vcf1 (1× – 30 ×).

**
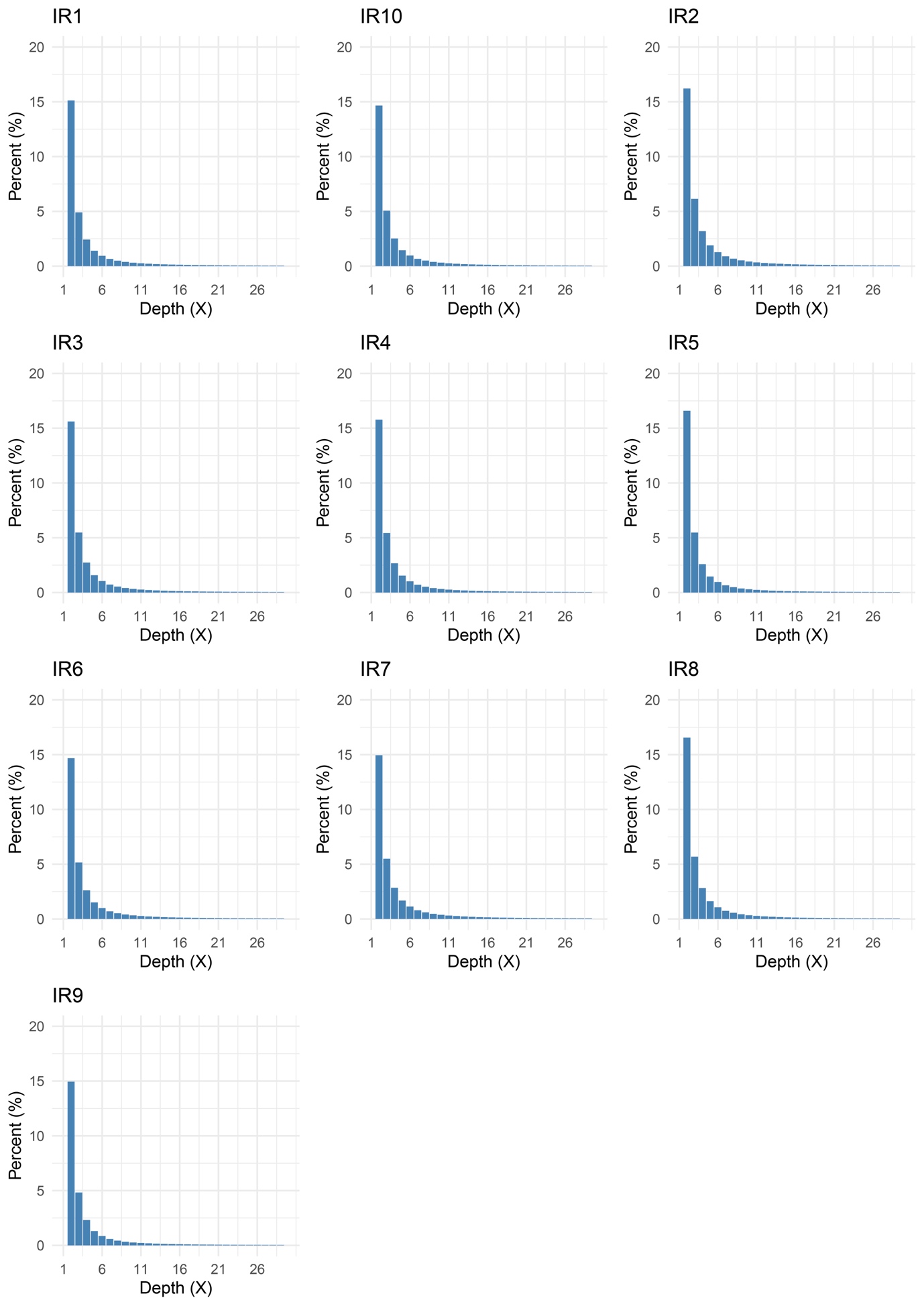
**

**Fig. S6** Summary of site depth for IN population based on vcf1 (1× – 30 ×).

**
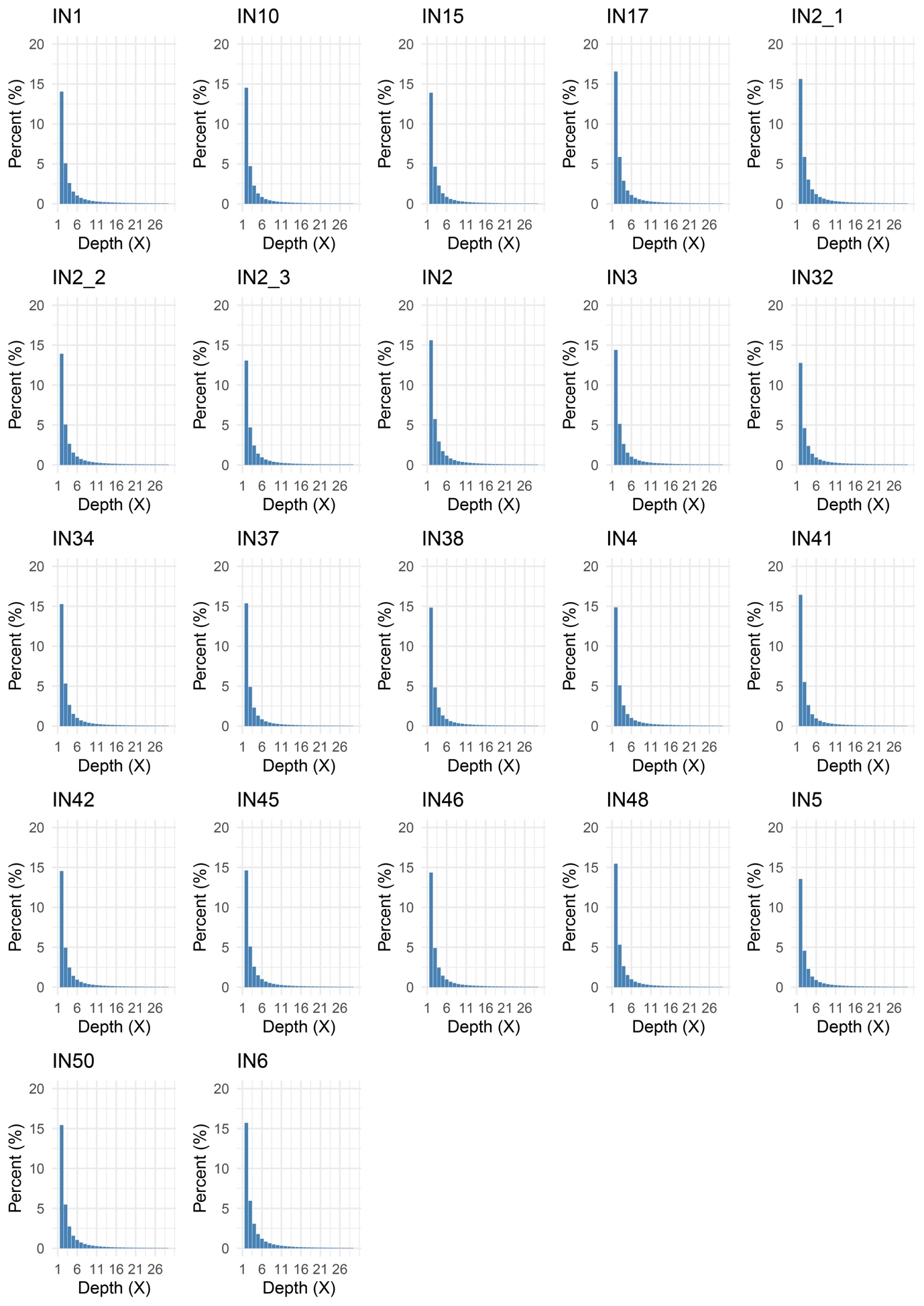
**

**Fig. S7** Summary of site depth for JR population based on vcf2 (1× – 200 ×).

**
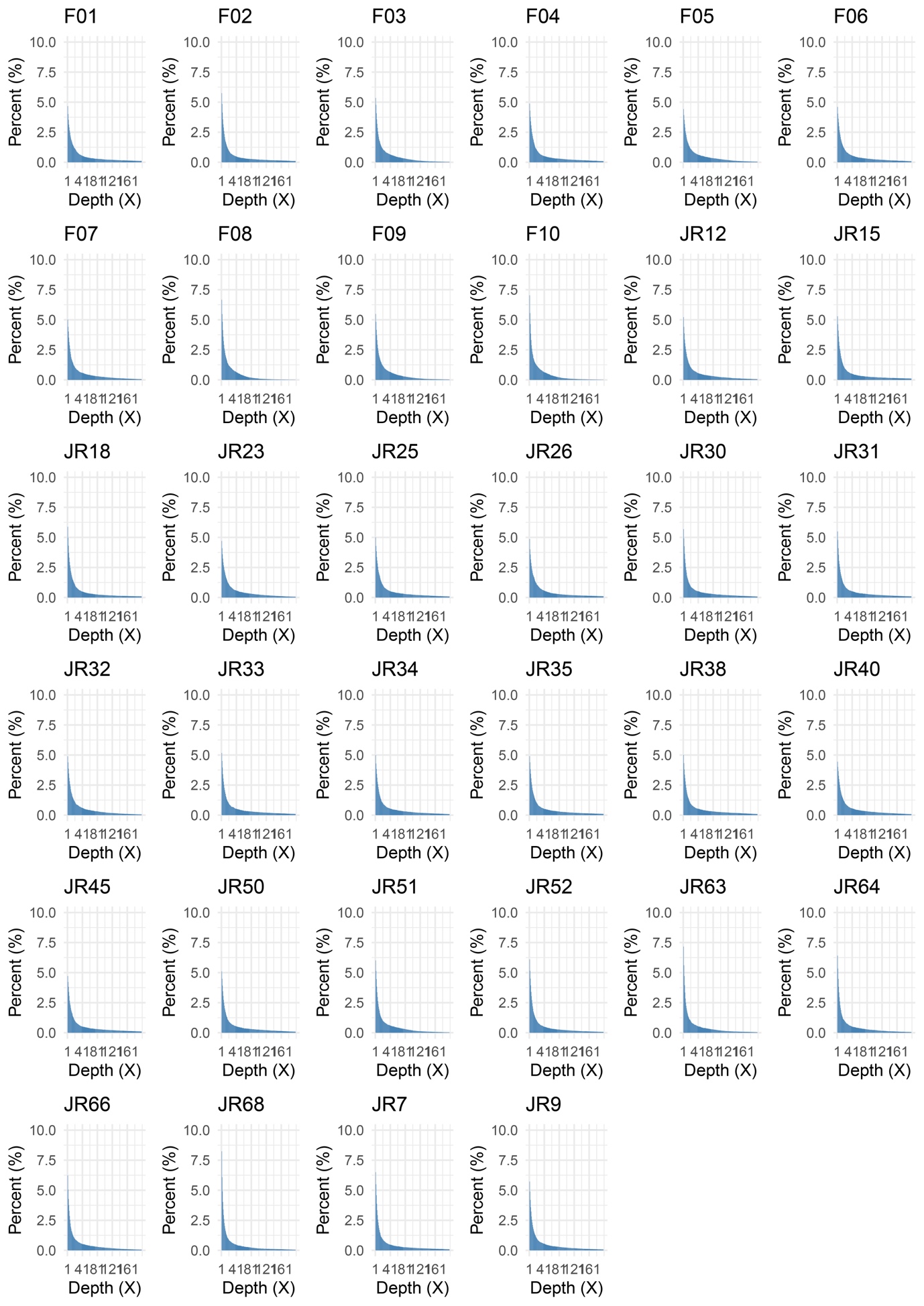
**

**Fig. S8** Summary of site depth for SB population based on vcf2 (1× – 200 ×).

**
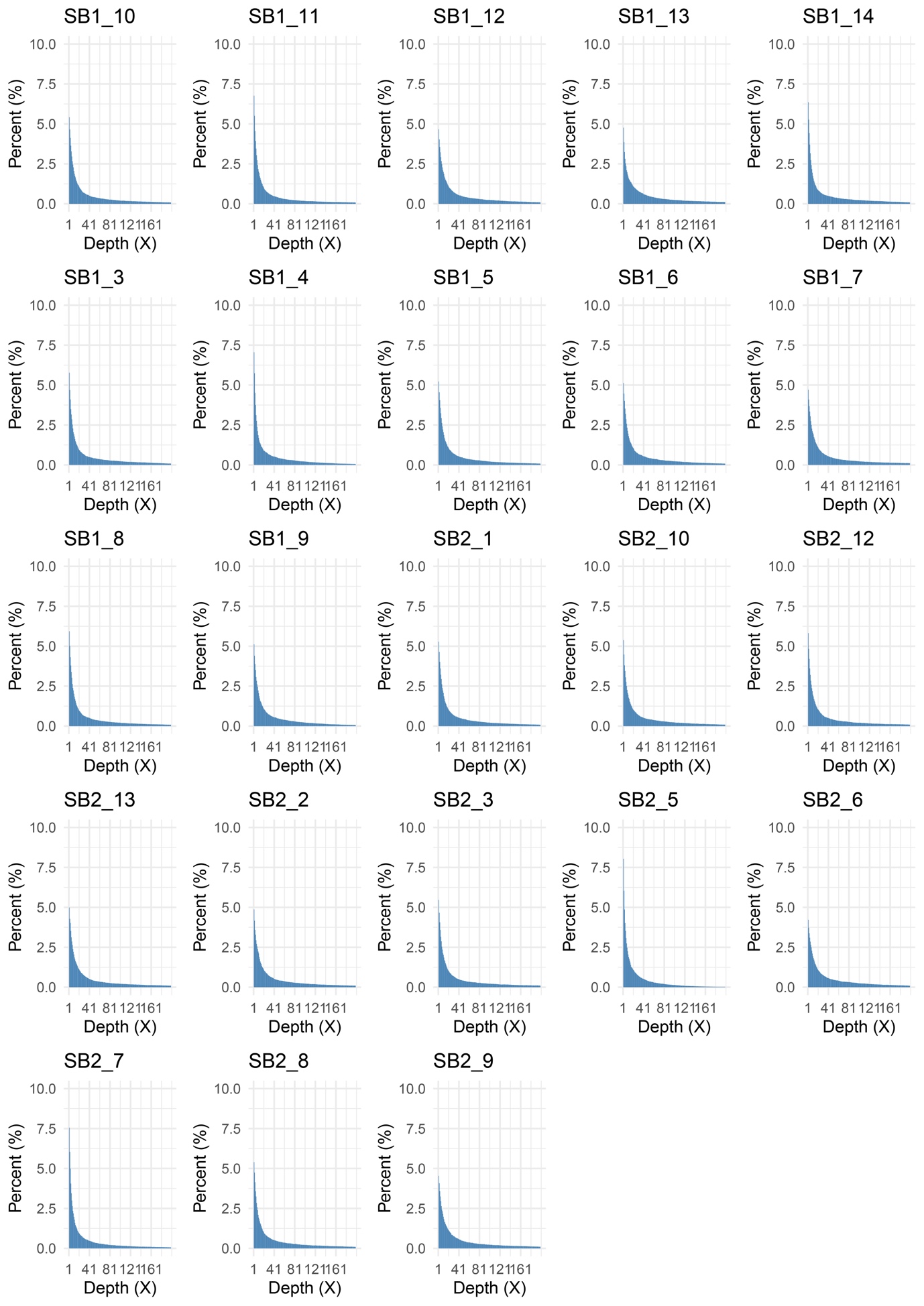
**

**Fig. S9** Summary of site depth for DK population based on vcf2 (1× – 200 ×).

**
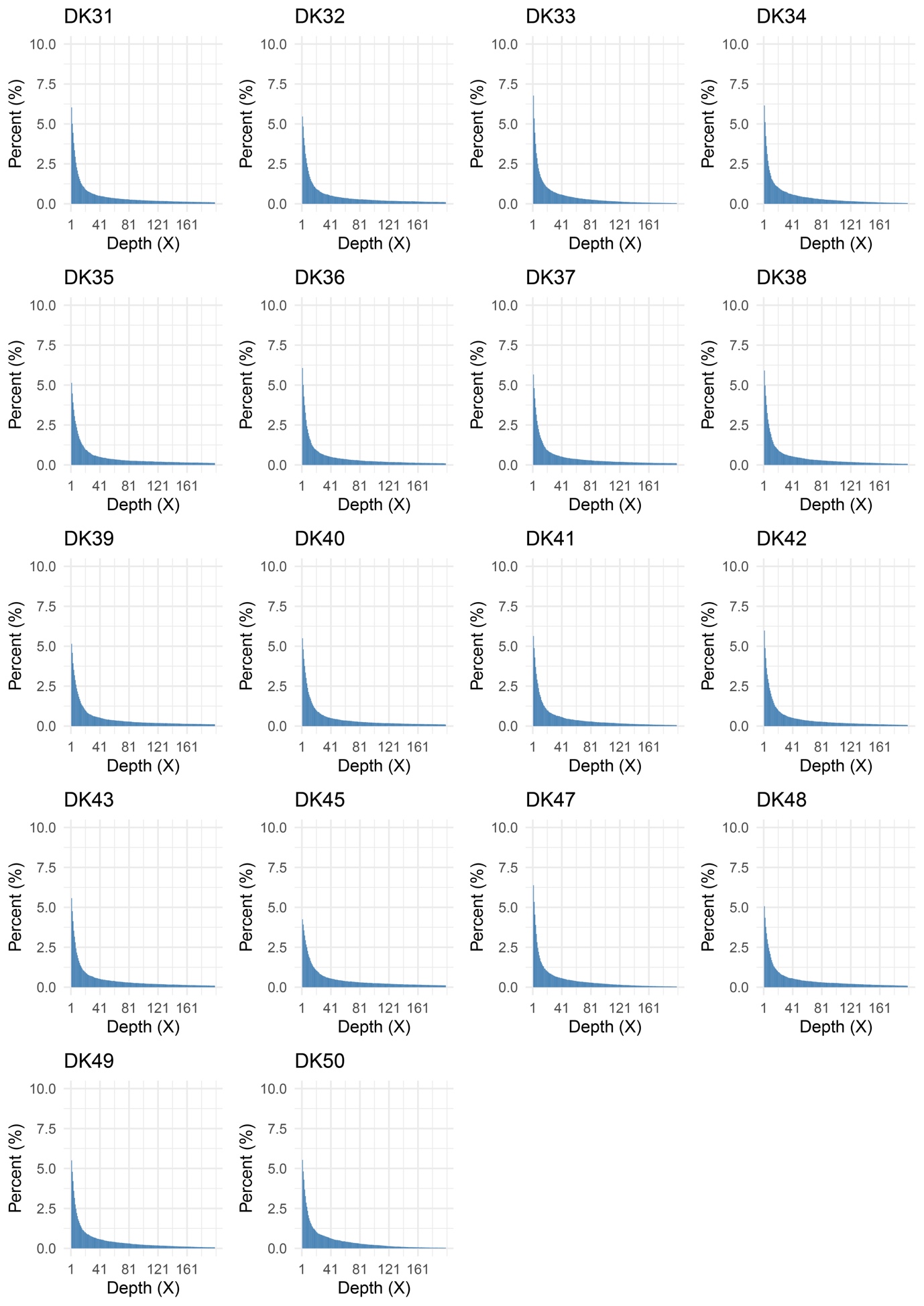
**

**Fig. S10** Summary of site depth for HK population based on vcf2 (1× – 200 ×).

**
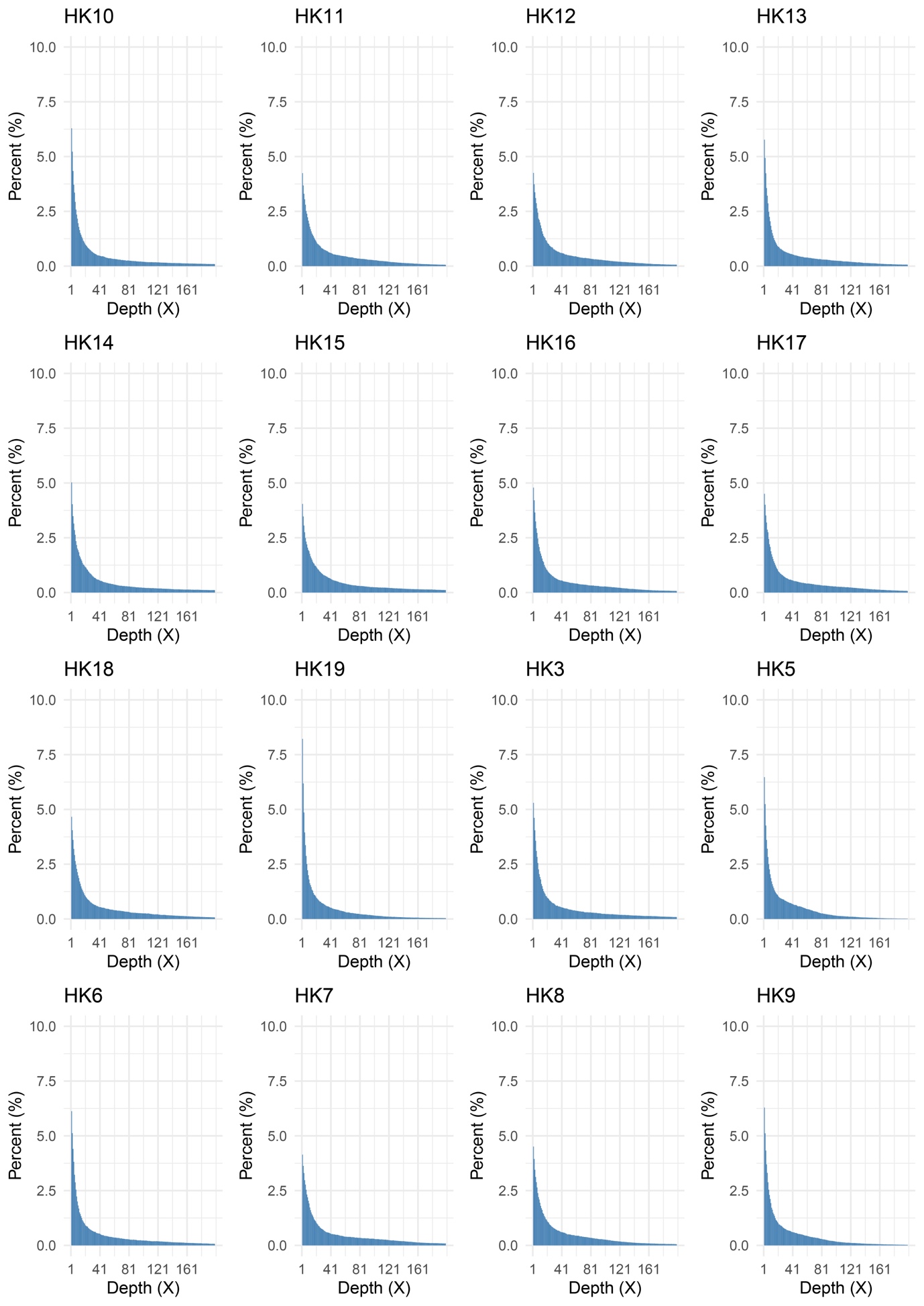
**

**Fig. S11** Summary of site depth for IR population based on vcf2 (1× – 200 ×).

**
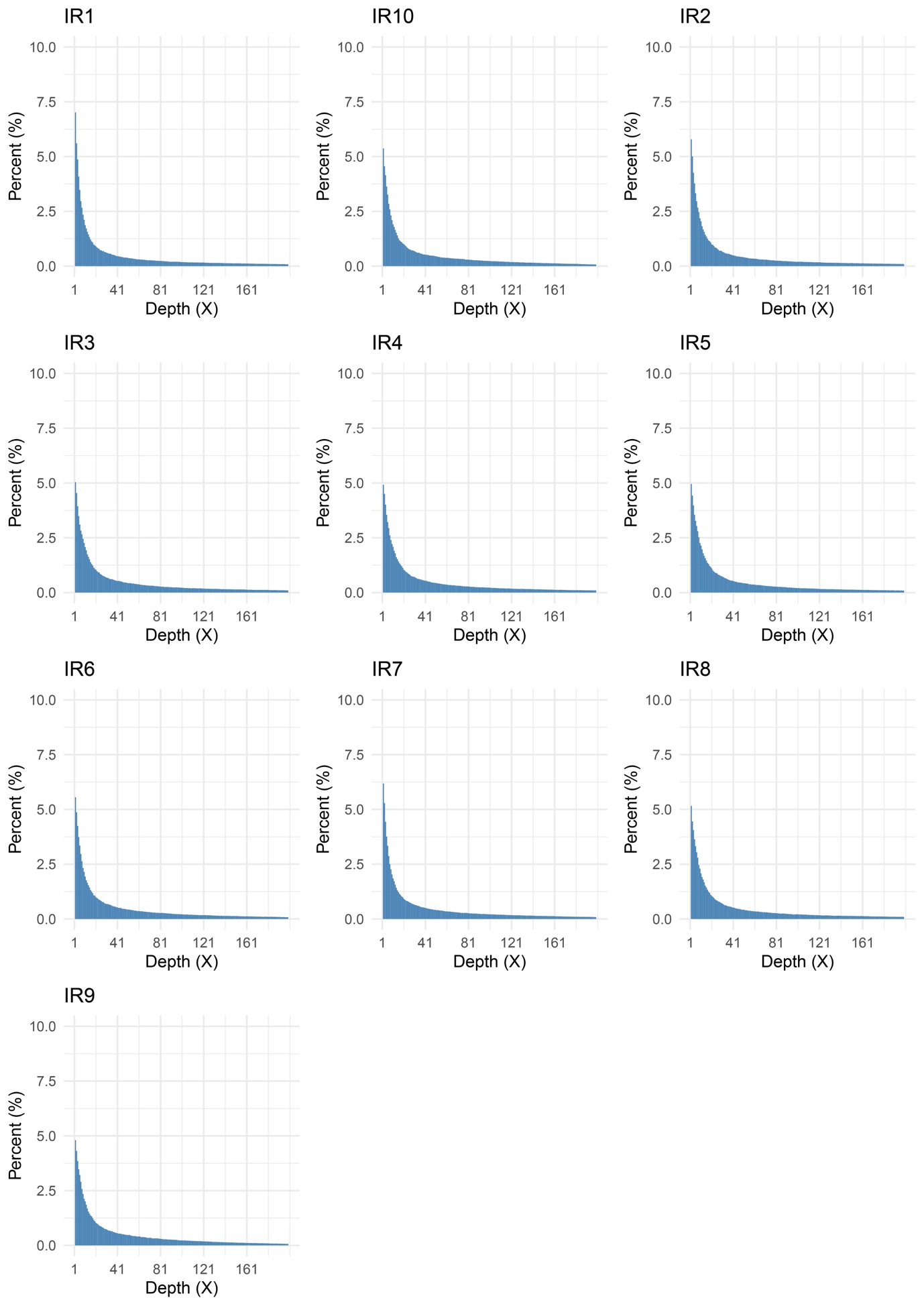
**

**Fig. S12** Summary of site depth for IN population based on vcf2 (1× – 200 ×).

**
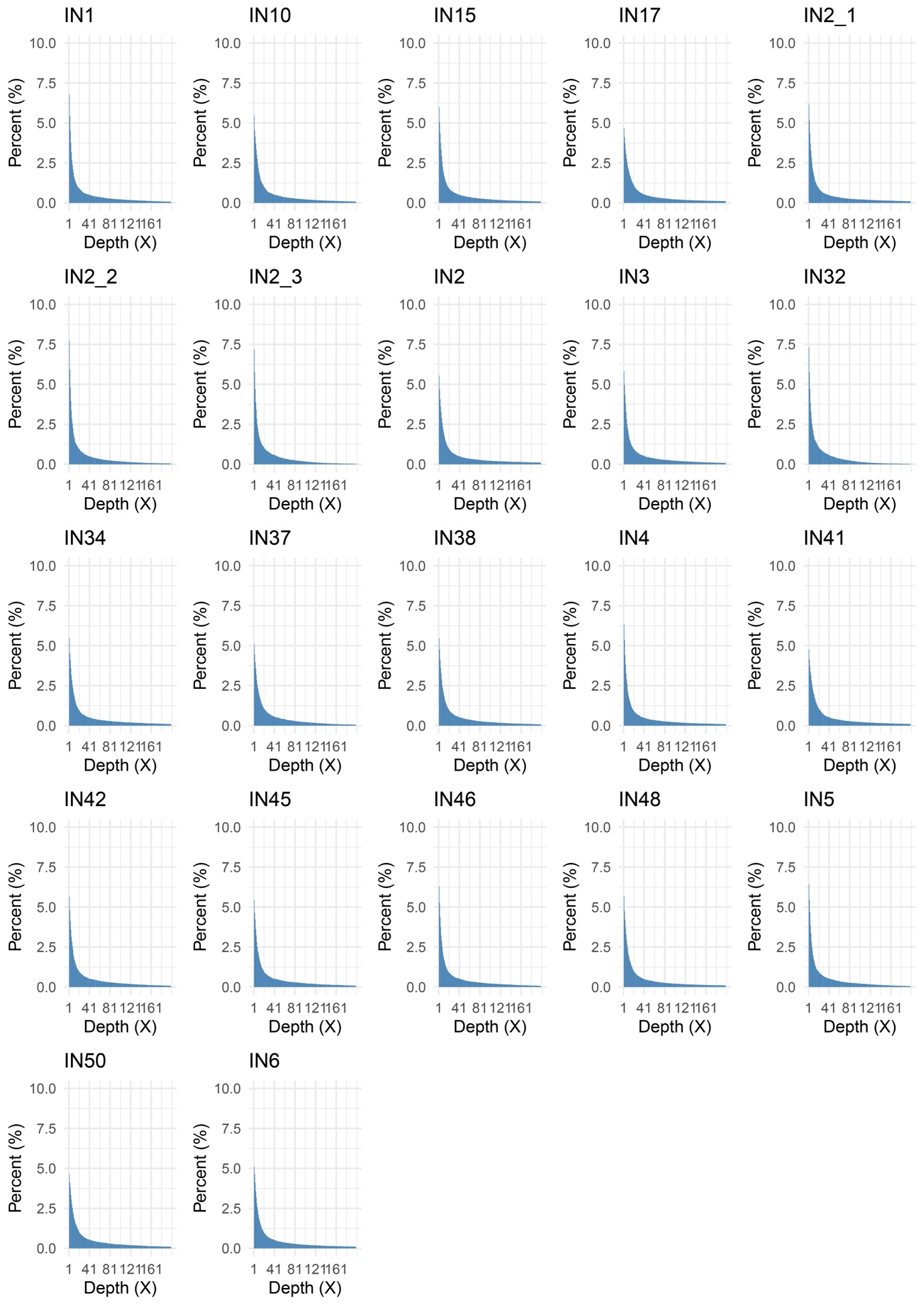
**

**Figure S13** Admixture results (K=2–4) for vcf1 (a) and vcf2 (b).


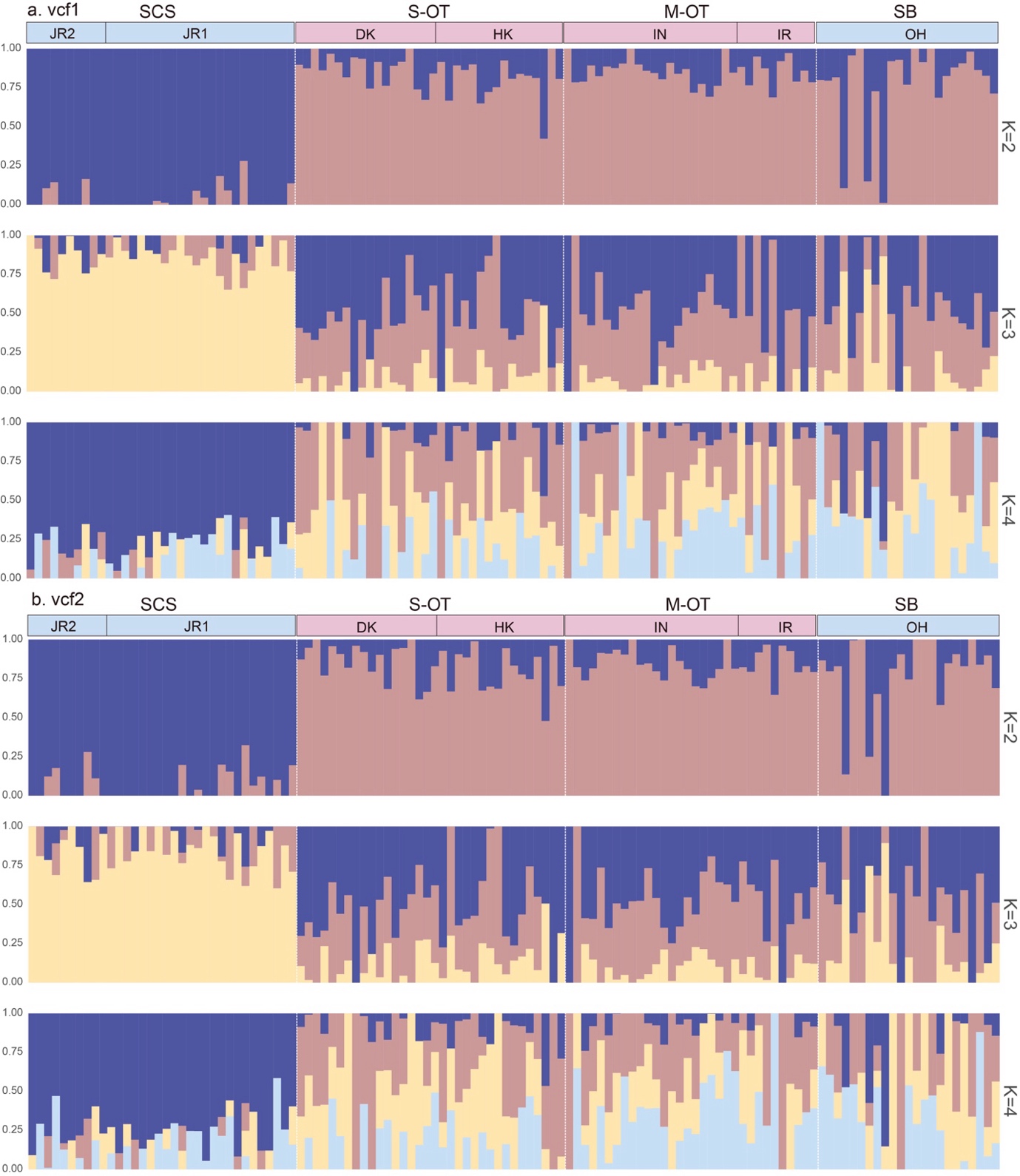


**Figure S14** DAPC result of vcf1 and vcf2 with K=2 and K=4.

**
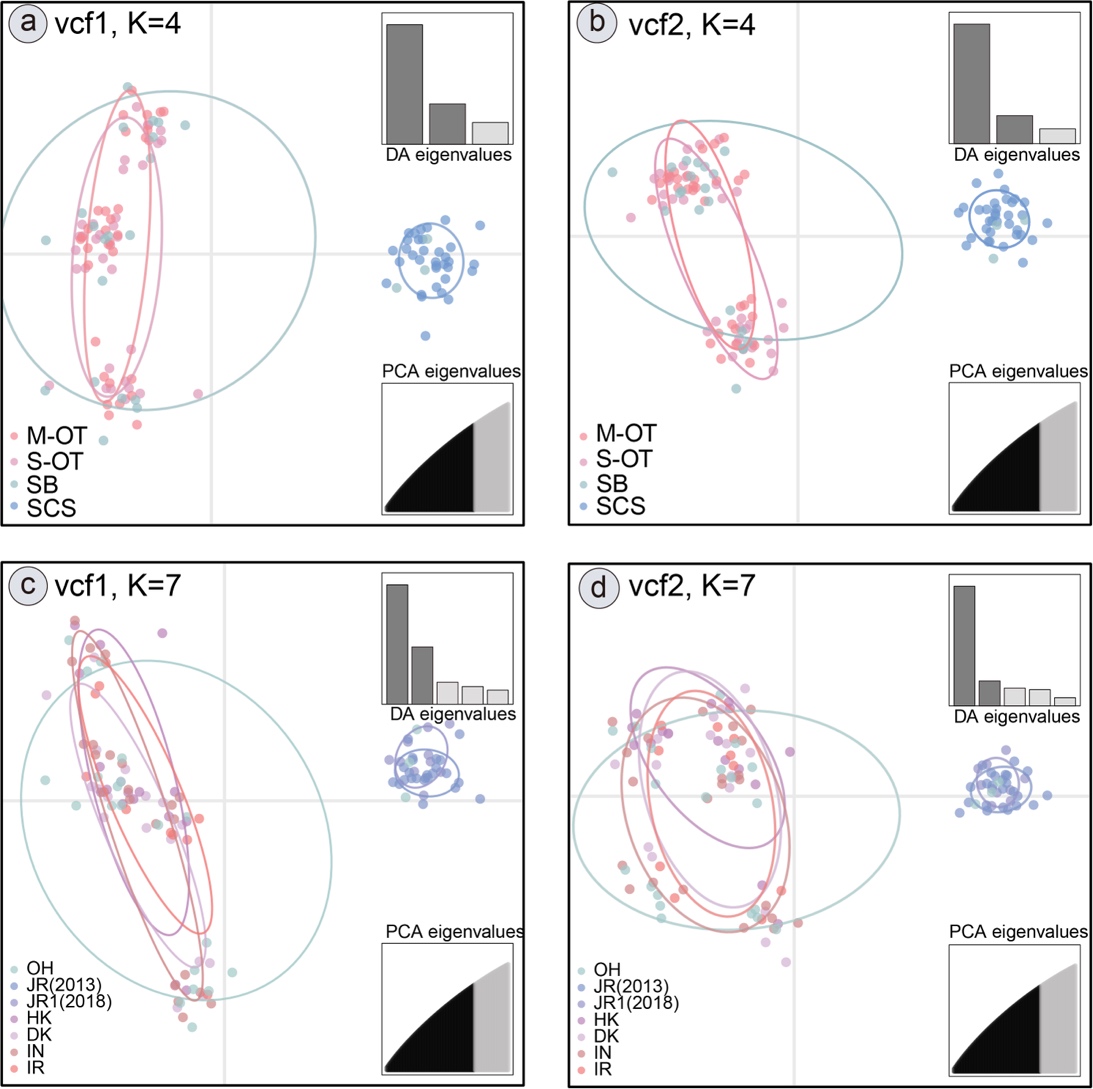
**

**Figure S15** Migration analysis by TreeMix using vcf1 (a) and vcf2 (b) with “m=0” setting.

**
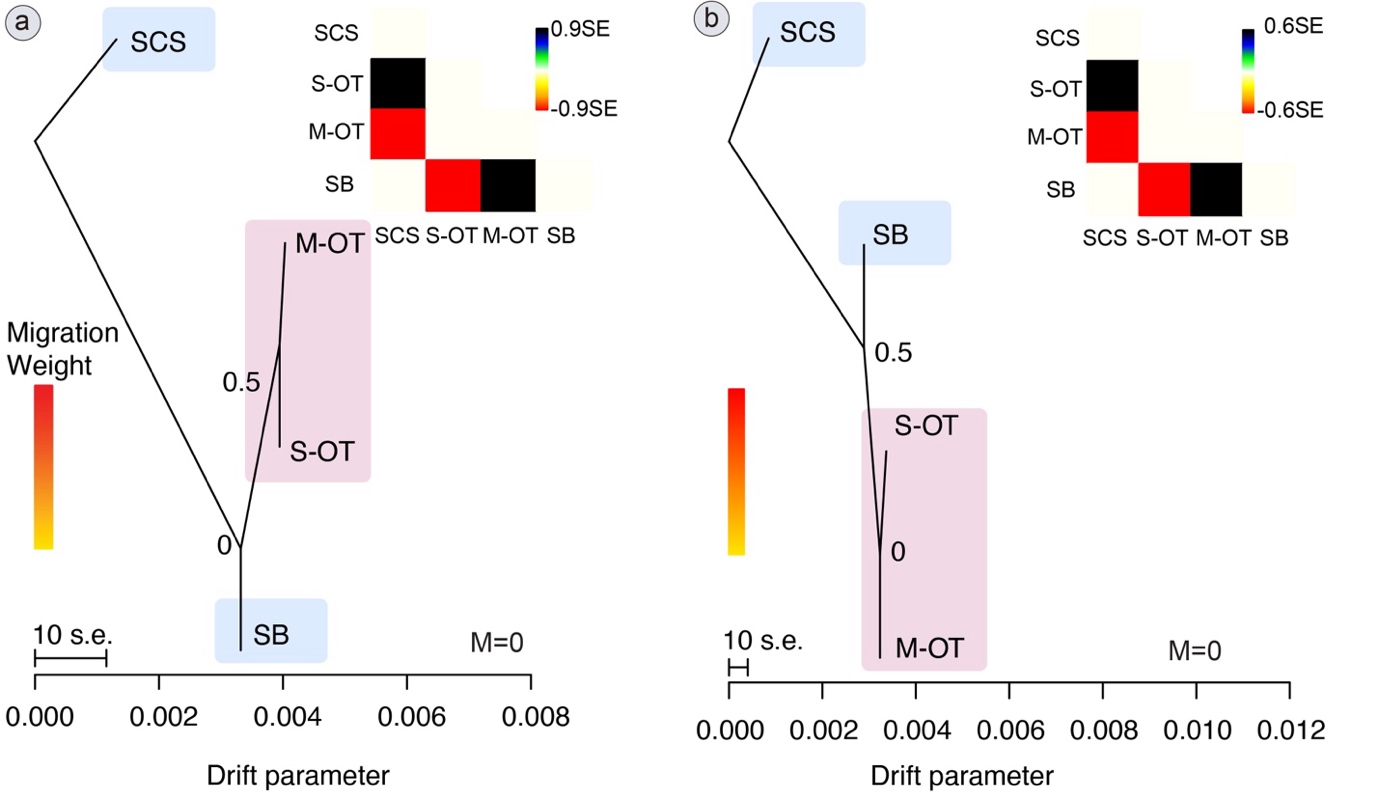
**

**Figure S16** Optimal migration edge analysis for vcf1 using OptM.


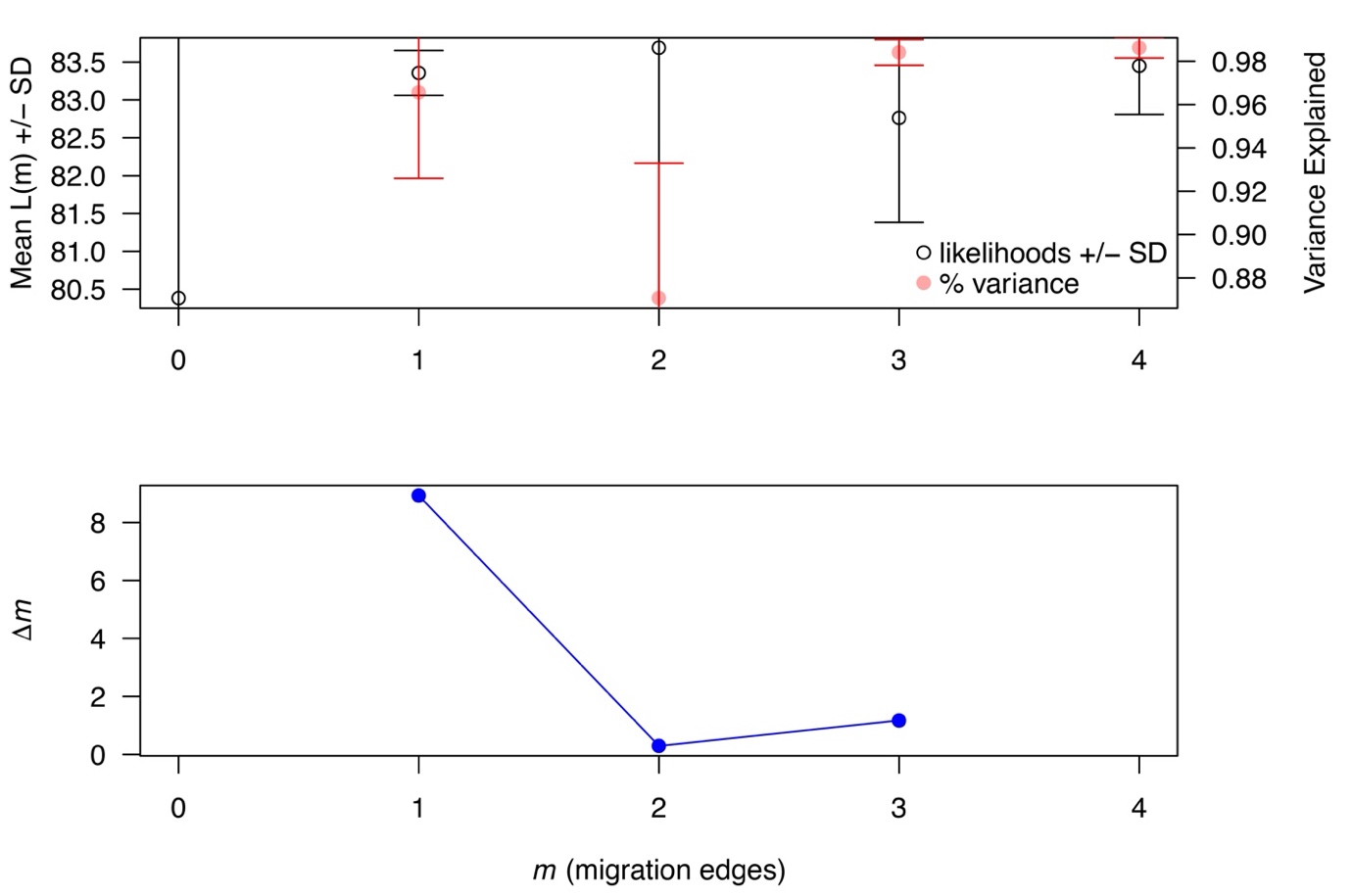


**Figure S17** Optimal migration edge analysis for vcf2 using OptM.


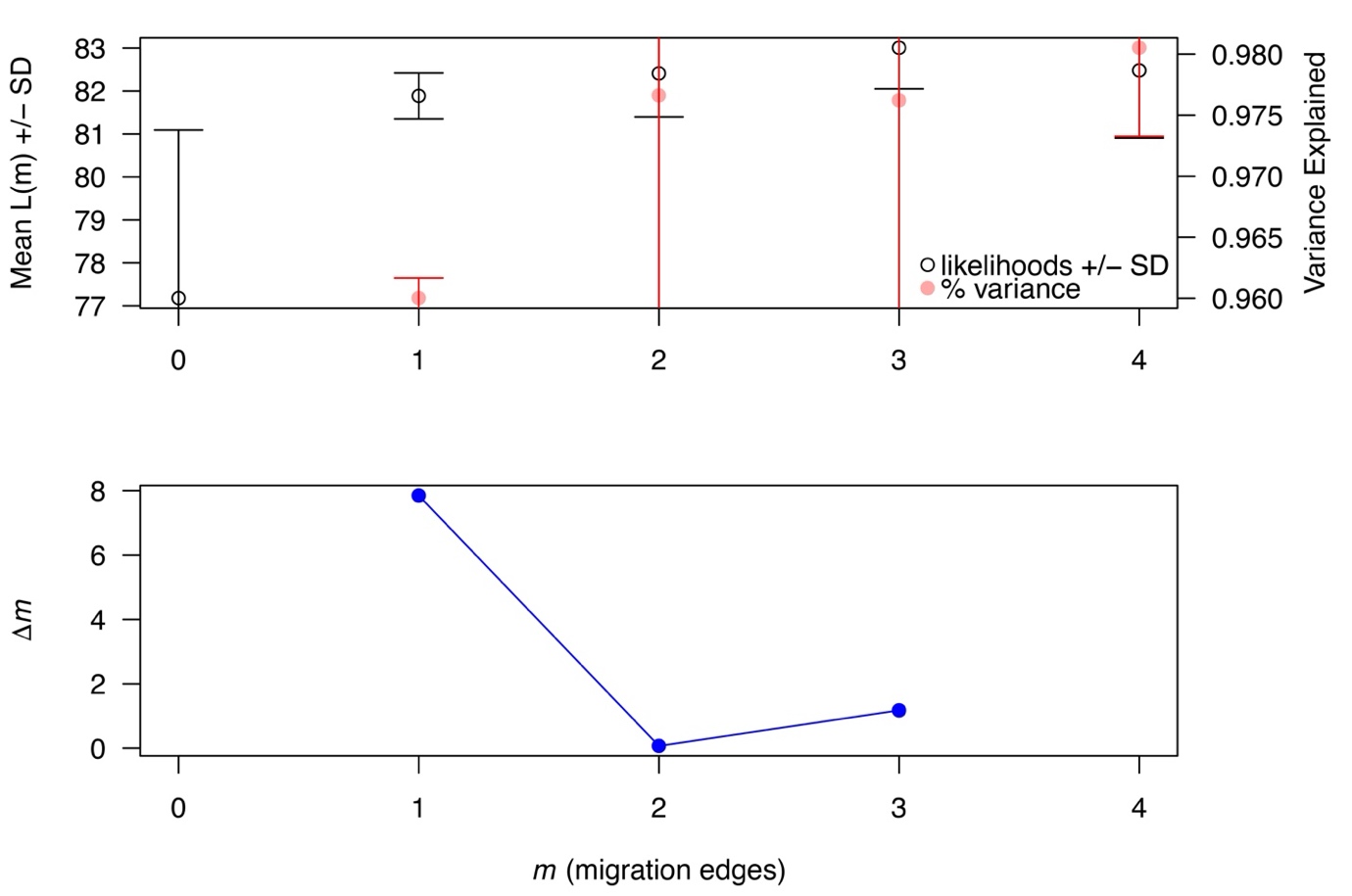


**Figure S18 BayeScan results using vcf1 dataset.** The number near black circle, site in vcf1 dataset.

**
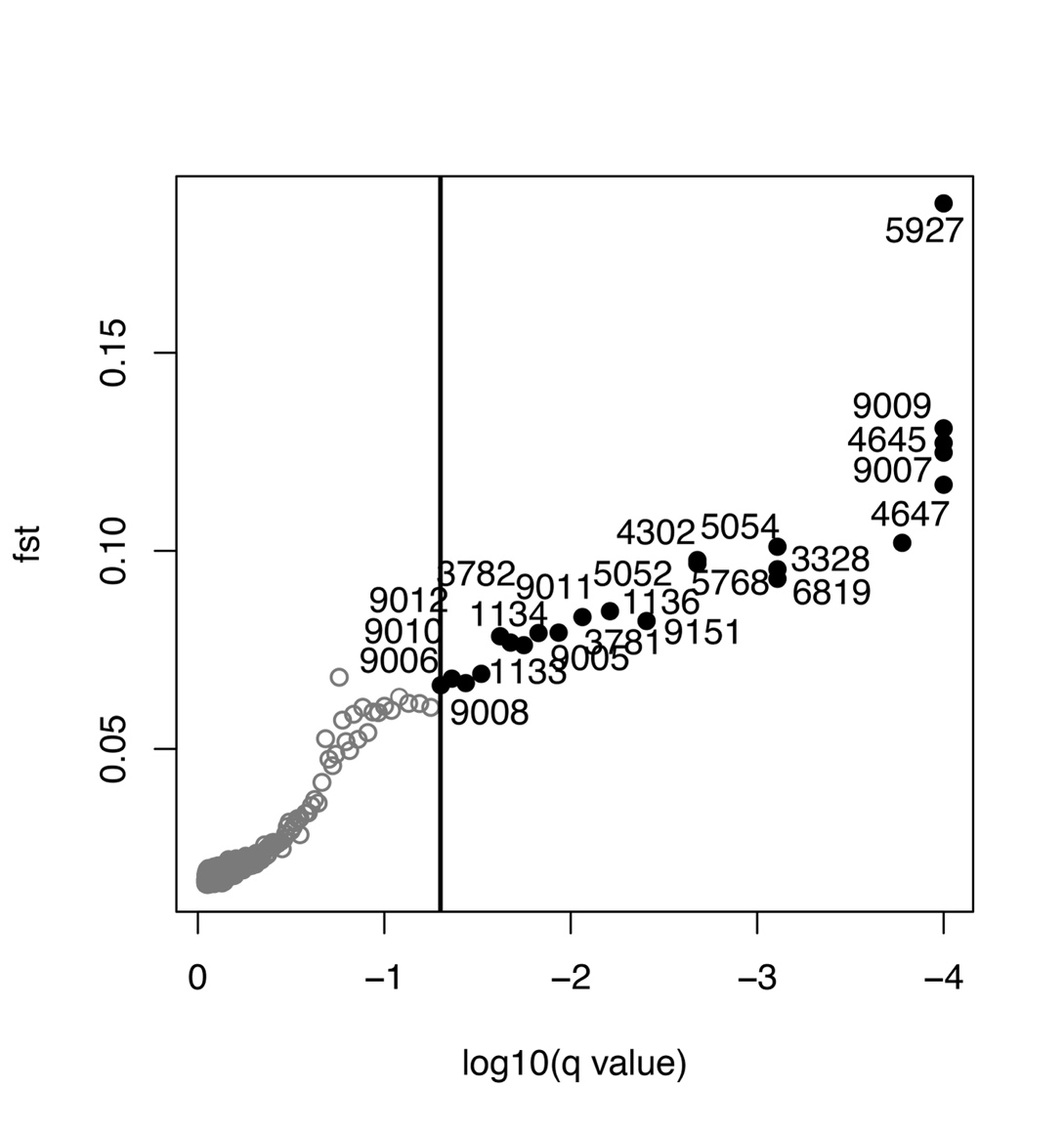
**

**Figure S19 BayeScan results using vcf2 dataset.** The number near black circle, site in vcf2 dataset.

**
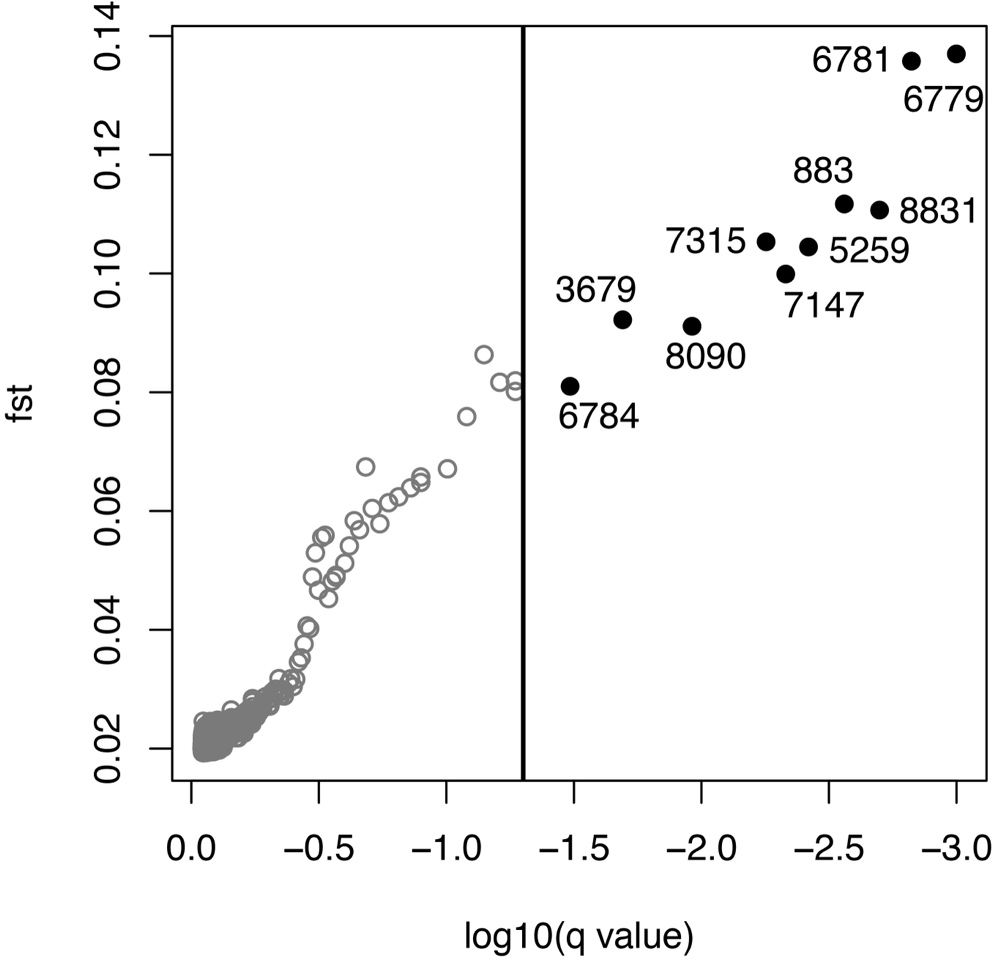
**

**Figure S20** *F_ST_* and π ratio analysis for individuals from seep and vent using vcf1 (a) and vcf2 (b). Pi_seep and Pi_vent, nucleotide diversity of the seep groups and the vent groups; Red data points located to the right of vertical dashed lines and above the horizontal dashed line, F_ST_ above 0.05 and in the top 10% right tail of the empirical nucleotide diversity distribution (Pi=1.48 for vcf1, Pi=1.28 for vcf2)

**
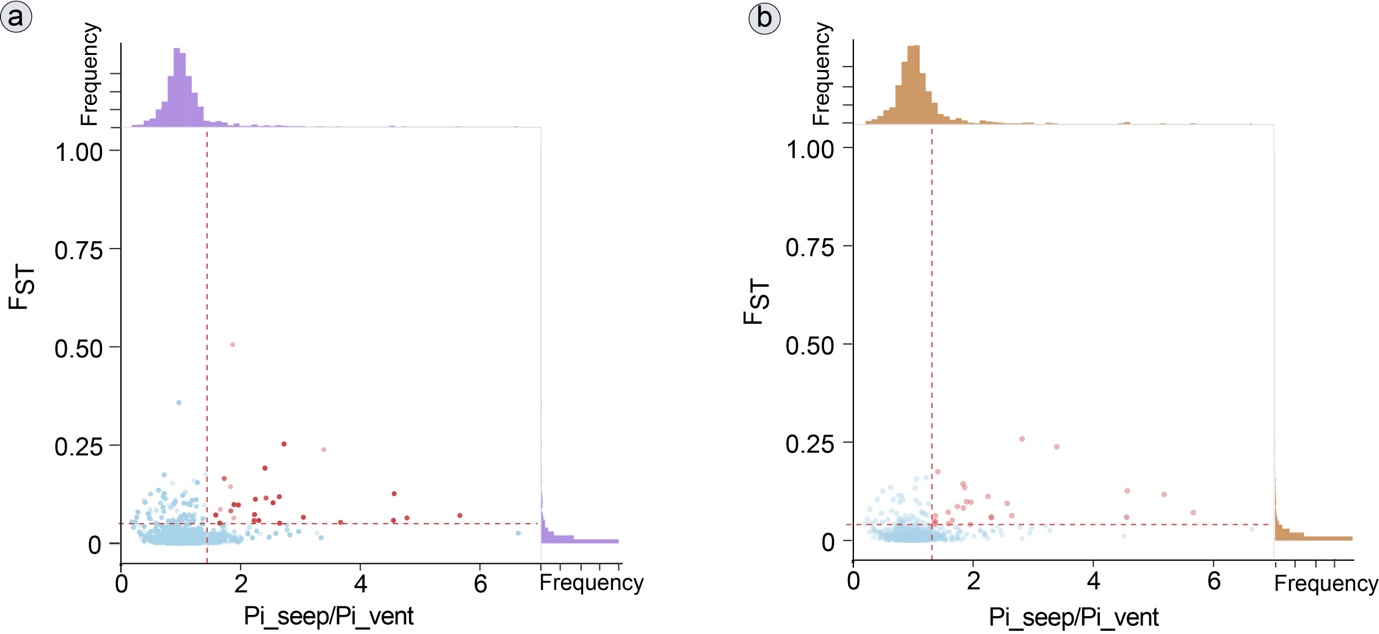
**

**Figure S21** *F_ST_* and π ratio analysis for individuals from SCS and OT-SB using vcf1 (a) and vcf2 (b). Pi_seep and Pi_vent, nucleotide diversity of the seep groups and the vent groups; Red data points located to the right of vertical dashed lines and above the horizontal dashed line, F_ST_ above 0.05 and in the top 10% right tail of the empirical nucleotide diversity distribution (Pi=1.48 for vcf1, Pi=1.28 for vcf2)


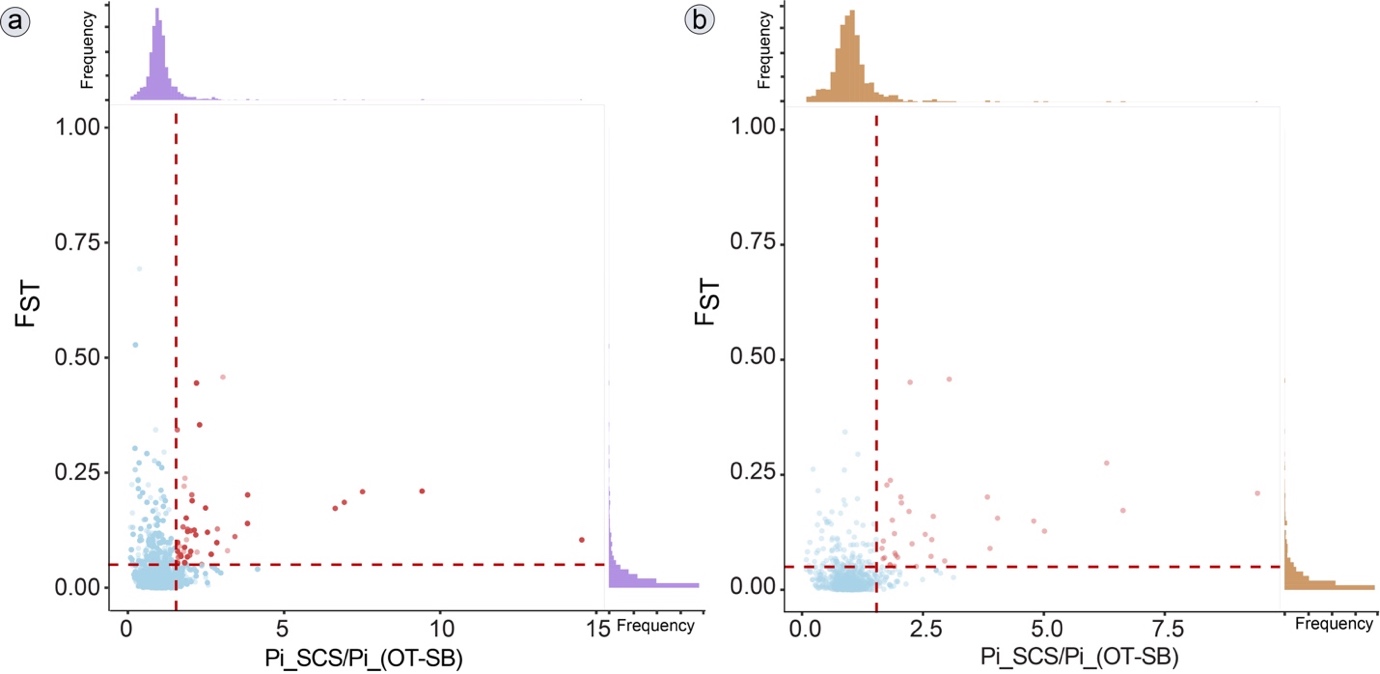


**Figure S22** F_ST_ and log-transformed π ratio analysis for individuals from seeps and vents using vcf1 (a) and vcf2 (b).


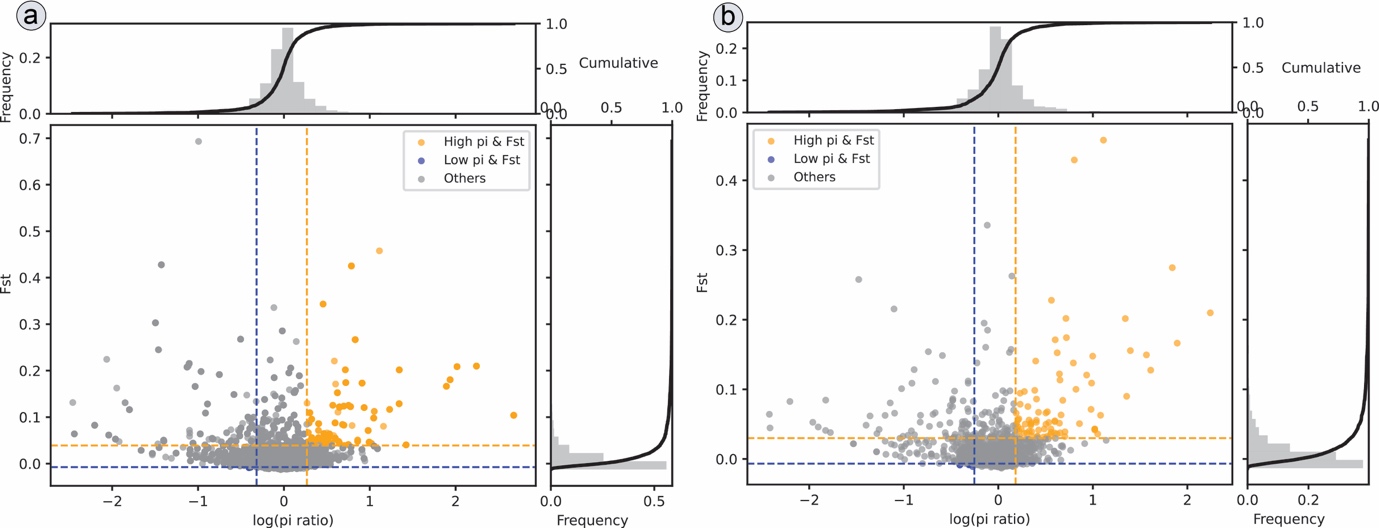


**Figure S23** PCA analysis for four linages (SCS, S-OT, M-OT, SB) using vcf3 (2064 SNPs, mapped with genome reference) and vcf4 (1811 SNPs, mapped with pseudoreference). Details of vcf3 and vcf4 in Table S16.


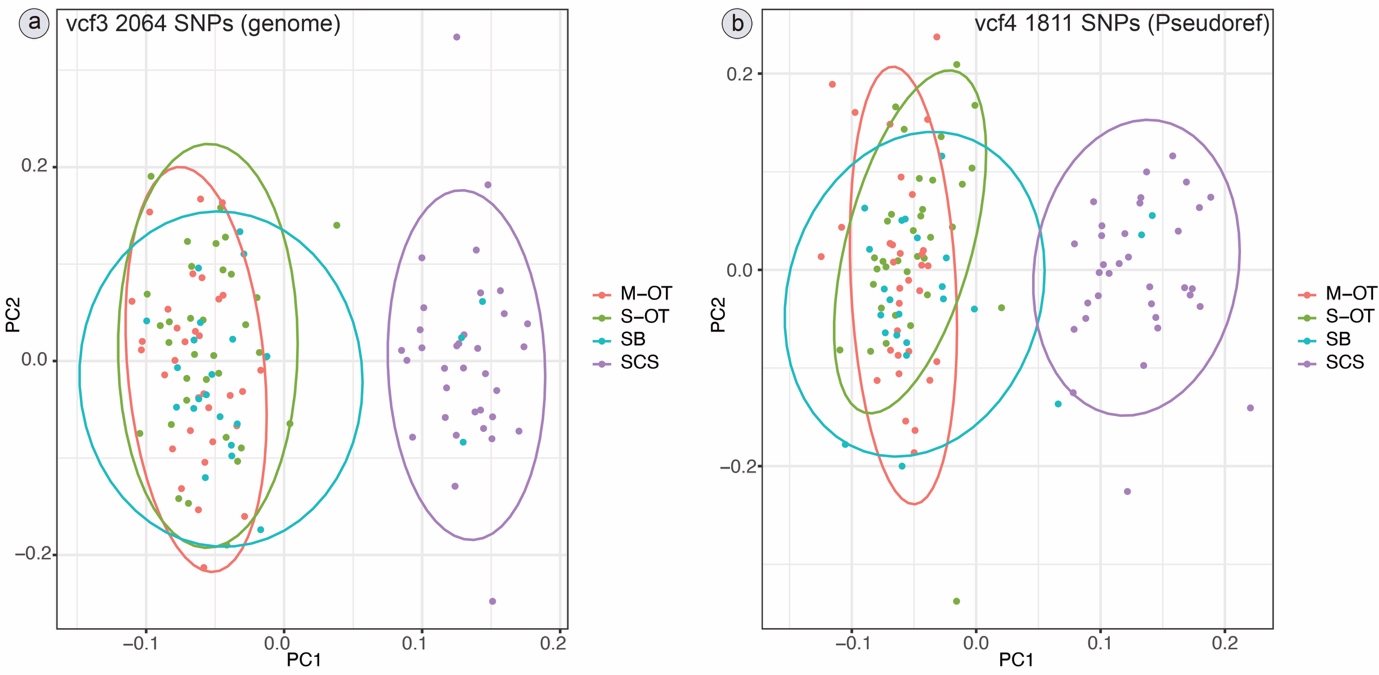

Supplement: Supplementary file 1 — Figure S1: Summary of site depth for JR population based on vcf1 (1× − 30×). Figure S2: Summary of site depth for SB population based on vcf1 (1× − 30×). Figure S3: Summary of site depth for DK population based on vcf1 (1× − 30×). Figure S4: Summary of site depth for HK population based on vcf1 (1× − 30×). Figure S5: Summary of site depth for IR population based on vcf1 (1× − 30×). Figure S6: Summary of site depth for IN population based on vcf1 (1× − 30×). Figure S7: Summary of site depth for JR population based on vcf2 (1× − 200×). Figure S8: Summary of site depth for SB population based on vcf2 (1× − 200×). Figure S9: Summary of site depth for DK population based on vcf2 (1× − 200×). Figure S10: Summary of site depth for HK population based on vcf2 (1× − 200×). Figure S11: Summary of site depth for IR population based on vcf2 (1× − 200×). Figure S12: Summary of site depth for IN population based on vcf2 (1× − 200×). Figure S13: Admixture results (K = 2–4) for vcf1 (a) and vcf2 (b). Figure S14: DAPC result of vcf1 and vcf2 with K = 2 and K = 4. Figure S15: Migration analysis by TreeMix using vcf1 (a) and vcf2 (b) with “m = 0” setting. Figure S16: Optimal migration edge analysis for vcf1 using OptM. Figure S17: Optimal migration edge analysis for vcf2 using OptM. Figure S18: BayeScan results using vcf1 dataset. The number near black circle, site in vcf1 dataset. Figure S19: BayeScan results using vcf2 dataset. The number near black circle, site in vcf2 dataset. Figure S20: F ST and π ratio analysis for individuals from seep and vent using vcf1 (a) and vcf2 (b). Pi_seep and Pi_vent, nucleotide diversity of the seep groups and the vent groups; Red data points located to the right of vertical dashed lines and above the horizontal dashed line, FST above 0.05 and in the top 10% right tail of the empirical nucleotide diversity distribution (Pi = 1.48 for vcf1 and Pi = 1.28 for vcf2). Figure S21: F ST and π ratio analysis for individuals from SCS and OT‐SB using vcf1 (a) [file EVA-19-e70195-s002.docx]
